# Se-Regulated MnS Porous Nanocubes Encapsulated in Carbon Nanofibers as High-Performance Anode for Sodium-Ion Batteries

**DOI:** 10.1007/s40820-025-01767-4

**Published:** 2025-04-28

**Authors:** Puwu Liang, Duo Pan, Xiang Hu, Ke R. Yang, Yangjie Liu, Zijing Huo, Zheng Bo, Lihong Xu, Junhua Xu, Zhenhai Wen

**Affiliations:** 1 State Key Laboratory of Structural Chemistry, and Fujian Provincial Key Laboratory of Materials and Techniques Toward Hydrogen Energy, Fujian Institute of Research on the Structure of Matter, Chinese Academy of Sciences, Fuzhou, 350002 People’s Republic of China; 2 College of Chemistry, Fuzhou University, Fuzhou, 350002 People’s Republic of China; 3 State Key Laboratory of Clean Energy Utilization, Zhejiang University, Hangzhou, 310027 People’s Republic of China; 4 Geological Survey of Finland, P.O. Box 96, 02151 Espoo, Finland; 5 Key Laboratory of Advanced Energy Materials Chemistry (Ministry of Education), Nankai University, Tianjin, 300071 People’s Republic of China

**Keywords:** Sodium-ion batteries, Anode, MnS_0.5_Se_0.5_, Carbon nanofiber, Defects

## Abstract

Manganese-based chalcogenides have significant potential as anodes for sodium-ion batteries (SIBs) due to their high theoretical specific capacity, abundant natural reserves, and environmental friendliness. However, their application is hindered by poor cycling stability, resulting from severe volume changes during cycling and slow reaction kinetics due to their complex crystal structure. Here, an efficient and straightforward strategy was employed to in-situ encapsulate single-phase porous nanocubic MnS_0.5_Se_0.5_ into carbon nanofibers using electrospinning and the hard template method, thus forming a necklace-like porous MnS_0.5_Se_0.5_-carbon nanofiber composite (MnS_0.5_Se_0.5_@N-CNF). The introduction of Se significantly impacts both the composition and microstructure of MnS_0.5_Se_0.5_, including lattice distortion that generates additional defects, optimization of chemical bonds, and a nano-spatially confined design. In situ/ex-situ characterization and density functional theory calculations verified that this MnS_0.5_Se_0.5_@N-CNF alleviates the volume expansion and facilitates the transfer of Na^+^/electron. As expected, MnS_0.5_Se_0.5_@N-CNF anode demonstrates excellent sodium storage performance, characterized by high initial Coulombic efficiency (90.8%), high-rate capability (370.5 mAh g^−1^ at 10 A g^−1^) and long durability (over 5000 cycles at 5 A g^−1^). The MnS_0.5_Se_0.5_@N-CNF //NVP@C full cell, assembled with MnS_0.5_Se_0.5_@N-CNF as anode and Na_3_V_2_(PO_4_)_3_@C as cathode, exhibits a high energy density of 254 Wh kg^−1^ can be provided. This work presents a novel strategy to optimize the design of anode materials through structural engineering and Se substitution, while also elucidating the underlying reaction mechanisms.

**Supplementary Information:**

The online version contains supplementary material available at 10.1007/s40820-025-01767-4.

## Highlights

- We have developed an efficient and scalable strategy to prepare a single-phase ternary MnS_0.5_Se_0.5_-carbon nanofiber composite with a defect-rich microstructure.
- The Na^+^ storage mechanism of manganese-based sulfides after the incorporation of Se was thoroughly investigated, through systematic electrochemical characterization combined with theoretical calculations.
- The MnS_0.5_Se_0.5_@N-carbon nanofiber composite, used as an anode, exhibits excellent reversible capacity, rate capability, and cycling stability in sodium-ion batteries.

**Supplementary Information:**

The online version contains supplementary material available at 10.1007/s40820-025-01767-4.

## Graphical abstract

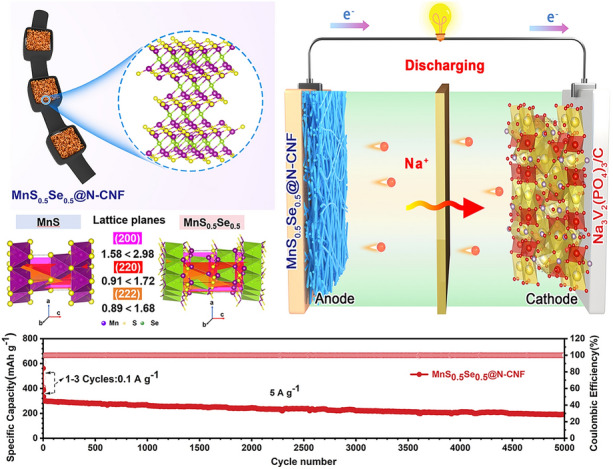

## Introduction

With the ever-increasing demand for electrically powered transportation vehicles, portable electronic devices, and renewable energy storage systems in modern society, the limited availability of lithium resources has posed significant challenges to the sustainable development of lithium-ion batteries (LIBs) for large-scale applications [1]. In contrast, sodium-ion batteries (SIBs) have emerged as a promising alternative to LIBs, due to the abundance of sodium (2.27% vs. lithium 0.002% of the Earth’s crust), low cost, and relatively high environmental friendliness [2]. However, the larger ionic radius of Na^+^ (0.102 nm vs. Li^+^ 0.076 nm) and its greater atomic mass (22.99 g mol^−1^ vs. Li^+^ 6.94 g mol^−1^) make it more difficult for sodium-ions to intercalate and de-intercalate in anode materials, which results in SIBs still facing challenges in energy density, power performance, and cycle life [3–5]. Therefore, continuous technological innovation and material optimization are expected to further enhance the sodium storage performance of SIB anode materials.

Transition metal chalcogenides (TMSs) are considered promising anode materials for SIBs due to their high theoretical capacity and good electrochemical activity [6]. Among them, manganese sulfide (MnS) has attracted significant attention due to its advantages, such as abundant reserves, low cost, and a high theoretical capacity of up to 616 mAh g^−1^ [7]. However, the critical problems of the large volume changes, inherently mediocre conductivity, slow Na^+^ reaction kinetics and high solubility of polysulfides during the continuous sodiation/desodiation process result in poor cyclability and inferior rate capability, which significantly hinders their practical applications [8–11]. In this regard, several strategies have been proposed to address these challenges: (i) the introduction of highly conductive carbon frameworks, which have proven effective in enhancing conductivity and providing structural support [12]. However, the poor interfacial binding between polar TMSs and non-polar carbon frameworks often leads to detachment of the active material from the carbon matrix during conversion reactions, severely affecting long-term cycle life [13]. (ii) Shrinking TMS particles to the nanoscale can effectively shorten the Na⁺ diffusion path and expose more active sites to improve reaction kinetics [14]. Unfortunately, the high specific surface energy of nanoscale materials often leads to severe aggregation [15]. (iii) Constructing a heterogeneous structure by combining different TMSs can form abundant phase boundaries and increase electrochemically active sites [16, 17]. Despite numerous efforts to improve electrochemical performance, the reaction kinetics during long-term cycling may still be hindered by the uneven distribution of heterogeneous interface and the gradual passivation of interfacial reactions [18].

It has been reported that introducing exogenous anions to form single-phase ternary metal dichalcogenide compounds (MX_a_X_1-a_, X = S, Se, Te…) is an effective strategy for modulating the electronic structure of TMSs [19]. The uniqueness of this approach lies in the ability to precisely regulate the electronic structure of MX_a_X_1-a_ at the atomic level [20, 21]. In particular, substituting Se as an anion in TMSs can significantly enhance their physicochemical properties. Firstly, Se, being in the same group as S, shares similar chemical properties, however, its atomic radius is smaller than that of S (1.98 vs. 1.84), and it has a smaller bandgap, which effectively enhances the electronic conductivity of TMSs and expands the lattice spacing [22]. Additionally, Se substitution creates new anion defect vacancies, promoting Na^+^ storage capacity [23]. The M-Se bond is weaker than the M-S bond, making it more likely to break during the conversion reaction, thus facilitating faster Na^+^ reaction kinetics [24]. Up to now, some MX_a_X_1-a_ anode materials have been reported, such as MoSSe@Rgo [25], CoS_2−*x*_Se_*x*_@SG [26], SnSe_0.5_S_0.5_@NG [27]. However, there are few reports on single-phase anion-doped manganese-based anodes with precisely controlled Se substitution content for SIBs [28, 29]. The relationship and mechanism between selenium doping/substitution content and electrochemical performance have not yet been sufficient clarified. Therefore, it is essential to further investigate the balance of electrochemical reactions in these materials.

Herein, we first synthesized manganese carbonate (MnCO_3_) nanocubes via the microemulsion precipitation method and mixed them with polyacrylonitrile. Subsequently, MnCO_3_ nanofibers were formed using the electrospinning technique. During the subsequent carbonization process, the decomposition of MnCO_3_ facilitated simultaneous sulfidation and selenization, enabling the controllable synthesis of MnS_0.5_Se_0.5_ porous nanocubes encapsulated in N-doped carbon nanofiber composite (MnS_0.5_Se_0.5_@N-CNF). The MnS_0.5_Se_0.5_ is firmly anchored in the carbon fibers through C–S–Mn and C–Se–Mn bonds, effectively alleviating volume expansion during cycling. The porous nanoscale MnS_0.5_Se_0.5_ cube not only prevent agglomeration of the active components, ensuring a short Na⁺ diffusion path, but also accelerate the transfer of ions and electrons. Experimental results and theoretical calculations demonstrate that Se substitution enhances the electronic conductivity of MnS and promotes Na⁺ diffusion kinetics. With these unique structural and compositional advantages, the MnS_0.5_Se_0.5_@N-CNF electrode exhibits excellent rate performance, high reversible capacity, and long-term cycling stability as the anode material for both sodium-ion half-cells and full cells.

## Experimental Section

### Chemicals

Manganese (II) Sulfate Monohydrate (MnSO_4_·H_2_O) were purchased from General Reagent. Sulfur sublimed (S), Cyclohexane (C_6_H_12_), 1-Butanol (C_4_H_10_O) and N,N-Dimethliformamide (C_3_H_7_NO) were purchased from Sinopharm Chemical Reagent Co., Ltd., China. Ammonium bicarbonate (NH_4_HCO_3_) were purchased from Macklin Reagent. Cetyltrimethylammonium (C_19_H_42_BrN) and Polyacrylonitrile (C_3_H_3_N)_n_) were purchased from Adamas beta. Selenium (Se) were purchased from Aladdin. All reagents and chemicals were of analytical grade and used without further purification.

### Synthesis

#### Synthesis of MnCO_3_ Nanocubes

In a typical synthesis process [30], 4 g of CTAB was dissolved in 100 mL of cyclohexane and 5 mL of n-butanol. After stirring evenly, 5 mL of 0.8 M NH_4_HCO_3_ aqueous solution was added. The mixed solution was stirred for 10 min until it became transparent. Then, 5 mL of 0.4 M MnSO_4_·H_2_O aqueous solution was continuously added drop by drop to obtain a milky white solution. Subsequently, the precipitate was obtained by centrifugation, washed several times with water and ethanol, and then dried in an oven at 60 °C to obtain white MnCO_3_.

#### Synthesis of MnCO_3_@PAN Nanofibers by Electrospinning

MnCO_3_@PAN was synthesized using a one-step electrospinning technique. Firstly, 1 g of MnCO_3_ powder was added into 5 mL of DMF. After ultrasonic treatment for 2 h, a homogeneous dispersion was obtained. Then, 800 mg of polyacrylonitrile (PAN) was added into the mixture. After stirring at 75 °C for 12 h, a uniform solution was obtained. The uniformly mixed solution was transferred into a 10 mL syringe connected with a stainless steel needle (model 15) for electrospinning and then installed in the electrospinning device. During the entire electrospinning process, a positive voltage of 16 kV and a negative voltage of 2 kV were applied to the needle tube, and it was operated at a propulsion speed of 0.015 mL min^−1^. The solution was ejected in the form of thin threads and collected on aluminum foil. The collected electrospun product was stabilized in air at 250 °C for 2 h.

#### Synthesis of MnS_0.5_Se_0.5_@N-CNF, MnS@N-CNF and MnSe@N-CNF Nanofibers

MnS_0.5_Se_0.5_@N-CNF was prepared by the simultaneous selenization and sulfidation method. Specifically, the precursor MnCO_3_@N-CNF and the mixture of selenium and sulfur powders (with a mass ratio of M precursor: M (ms_e_:ms = 1.51:1) = 1:2) were placed at the downstream and upstream of the quartz tube, respectively. Then, in a hydrogen-argon mixed atmosphere at 500 °C, the selenization/sulfidation was carried out for 2 h with a heating rate of 5 °C min^−1^. In addition, the processes of adding only sulfur source or selenium source separately were the same as those for prepar MnS_0.5_Se_0.5_@N-CNF. Finally, the obtained samples were named MnS@N-CNF and MnSe@N-CNF, respectively.

### Characterization

Analyzing the microstructure and morphology of samples by field emission scanning electron microscopy (FESEM, Hitachi SU-8020), field emission transmission electron microscopy (TEM, Tecnai G2 F20 S-TWIN TMP) and high-resolution transmission electron microscopy (HRTEM). Energy dispersive X-ray (EDX) spectroscopy was used to determine the distribution of elements. The crystalline phase structure of the sample was tested by X-ray powder diffractometer (XRD, Miniflex600 powder X-ray diffractometer with Cu Kαradiation). The Raman spectra were obtained by confocal Raman spectroscopy with a 532 nm light source (LabRAM HR). Nitrogen adsorption/desorption isotherms from the Automatic Specific Surface and Porosity Analyzer (Micromeritics ASAP 2460). The defective condition of the sample was shown by the paramagnetic resonance spectrometer (EPR, Bruker-E500). The form of the chemical state in which the element exists is illustrated by the X-ray photoelectron spectrometer (XPS, Thermo Scientific K-Alpha). The carbon content of the samples was obtained by a comprehensive thermal analyzer (TGA, NETZSCH STA449F3) in a flow of air with a heating rate of 10 °C min^−1^. To characterize the ex-situ XPS and TEM tests during the first cycle, the cells at different cut-off voltages were carefully disassembled inside an argon-filled glove box and the electrodes were washed in diethylene glycol dimethyl ether solvent for several times to remove residual electrolyte.

### Electrochemical Measurements

The electrochemical testing was performed by assembly standard 2032 typed coin cells in an argon-filled glove box (< 0.01 ppm of moisture and oxygen contents). For the half-cell, the prepared active materials were used as the cathode, a homemade sodium metal sheet was used as anode, the electrolyte was 1.0 M NaPF_6_ dissolved in diethylene glycol dimethyl ether (DIGLYME) and a glass fiber filter paper (GF/D, Whatman) was used as the separator. Preparation of the working electrode consists of a homogeneous slurry of containing active material (80 wt%), conductive carbon black (10 wt%) and sodium carboxymethyl cellulose (10 wt%) mixed in deionized water scraped and coated on the copper foil collector, subsequent drying in a 70 °C oven overnight. The active material loading mass approximately 0.8–1.0 mg cm^−2^. Galvanostatic charging/discharging tests on a multi-channel LAND battery test system (Wuhan, China) with a potential range of 0.01–3.00 V at room temperature. Cyclic voltammetry (CV) curves were obtained using a CHI660E electrochemical workstation, and electrochemical impedance spectroscopy (EIS) was performed over a frequency range from 0.01 Hz to 100 kHz. The galvanostatic intermittent titration technique (GITT) tests were conducted under constant-current conditions at 0.05 A g^−1^ for 20 min, followed by rest intervals of 30 min after the third cycles.

For the construction of the MnS_0.5_Se_0.5_@N-CNF//Na_3_V_2_(PO_4_)_3_@C sodium-ion full cell, the cathode was fabricated through a specific process. A slurry was prepared by mixing Na_3_V_2_(PO_4_)_3_@C (NVP@C), conductive carbon black, and poly (vinylidene fluoride) (PVDF) in a weight ratio of 8:1:1 in N-methylpyrrolidone (NMP) solvent. This slurry was then cast onto aluminum current collectors and dried under vacuum at 80 °C overnight. The MnS_0.5_Se_0.5_@N-CNF electrode, after undergoing electrochemical activation to eliminate initial and irreversible capacity, functions as the anode. In all electrochemical tests, the electrolyte was 1.0 M NaPF_6_ dissolved in DIGLYME for the full cell and NVP@C half-cell. It is remarkable that the cycling specific capacity of NVP@C at 0.1 A g^−1^ averages around 90 mAh g^−1^. Therefore, to ensure a balanced capacity between the cathode and anode, the cathode/anode active mass ratio in the MnS_0.5_Se_0.5_@N-CNF// NVP@C full cell is precisely controlled to be approximately 5:1. The full cells were evaluated within a voltage range of 1.0 to 3.8 V in a multi-channel LAND battery test system at room temperature. The separator and the electrolyte utilized in the full cells were exactly the same as those employed in the half-cells.

## Results and Discussion

### Synthesis and Characterization of Samples

**Fig. 1:**
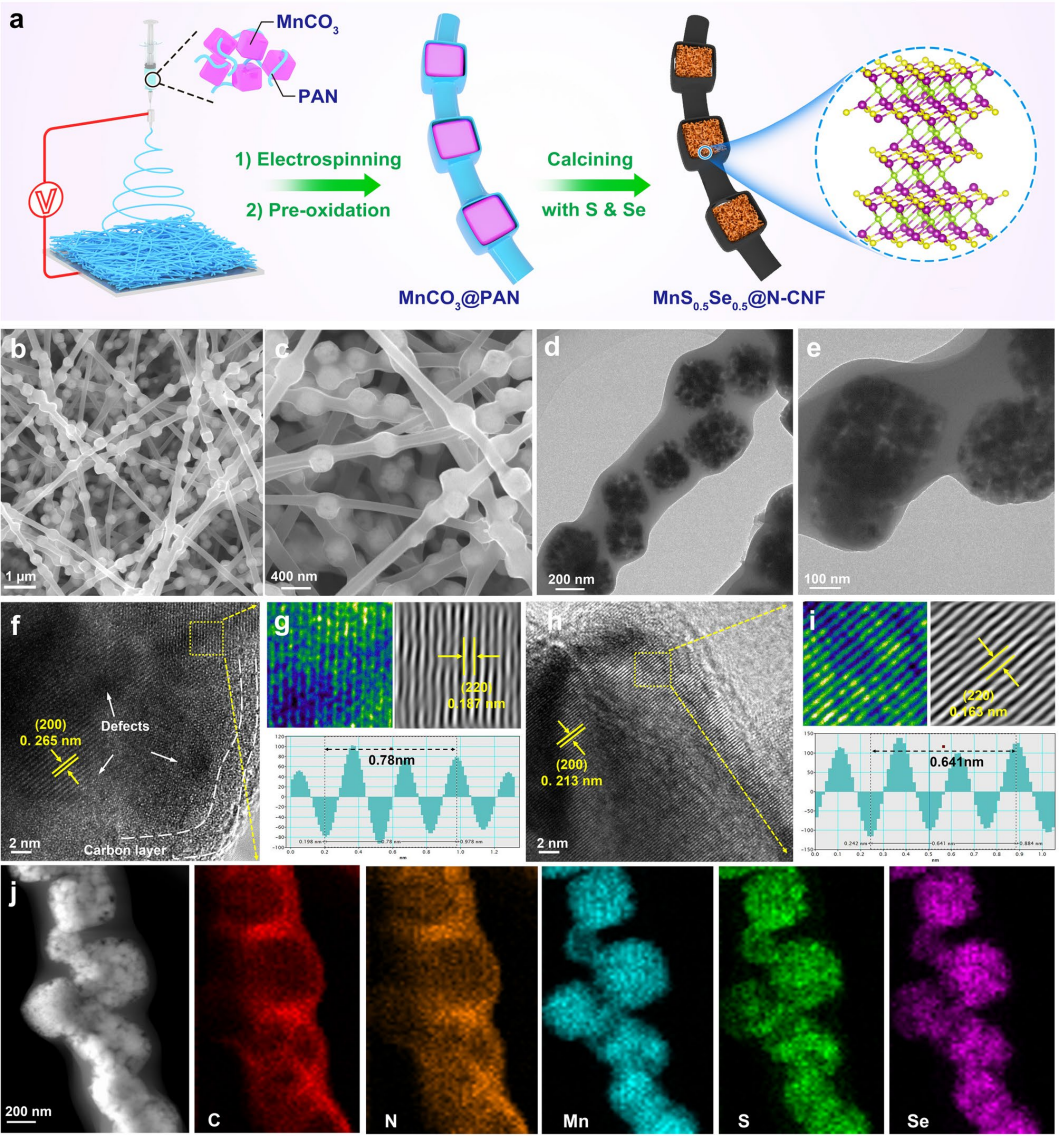
**a** Schematic strategy of synthesis for the MnS_0.5_Se_0.5_@N-CNF composites, **b**, **c** FESEM images and **d**, **e** TEM images of MnS_0.5_Se_0.5_@N-CNF, **f**, **g** and **h, i** HRTEM image and related lattice stripe marking images with FFT-filtered of MnS_0.5_Se_0.5_@N-CNF and MnS@N-CNF, **j** EDS elemental mappings of MnS_0.5_Se_0.5_@N-CNF composite

The synthesis process of MnS_0.5_Se_0.5_@N-CNF composites is schematically illustrated in Fig. 1a. Initially, highly uniform MnCO_3_ nanocubes were prepared by the microemulsion precipitation method, exhibiting regular cubic shapes with side lengths of approximately 200 nm (Fig. S1a-c). Next, after being added to a DMF solution containing PAN, the MnCO_3_ nanocubes were aligned in series by the electrospinning method, forming a unique nanofiber structure resembling a “necklace”. Finally, during the carbonization process, the solid MnCO_3_ nanocubes underwent thermal decomposition while simultaneously undergoing selenization and sulfidation. This process led to the formation of hollow porous MnS_0.5_Se_0.5_ nanocages, which were embedded in and strung together by carbon nanofibers to obtain the “necklace”-shaped MnS_0.5_Se_0.5_@N-CNF composite.

The field emission scanning electron microscopy (FESEM) and TEM images of the MnS_0.5_Se_0.5_@N-CNF reveal a three-dimensional network structure where carbon nanofibers are interlaced and crossed in a staggered manner, with nanocubes uniformly arranged within the carbon nanofibers (Fig. 1b–d). The magnified TEM image further shows that the MnS_0.5_Se_0.5_@N-CNF exhibits hollow porous nanocages encapsulated in carbon nanofibers, which effectively prevents the direct exposure of the MnS_0.5_Se_0.5_ nanocages to the electrolyte and enhance the conductivity of the composite (Fig. 1e) [31]. The selected-area electron diffraction (SAED) pattern displays multiple diffraction rings (Fig. S2), indicating the polycrystalline characteristics of MnS_0.5_Se_0.5_@N-CNF nanostructures. These diffraction rings can be indexed to the (200), (220), (222), and (400) crystal planes of MnS_0.5_Se_0.5_ [32]. The high-resolution TEM (HRTEM) image shows a parallel stacking structure with a large interlayer spacing of 0.265 nm corresponding to the (200) plane, and clearly reveals a carbon layer wrapping the surfaces of the MnS_0.5_Se_0.5_ nanocages (Fig. 1f). Additionally, lattice fringes with a spacing of 0.187 nm are observed, aligning well with the (220) plane of MnS_0.5_Se_0.5_. More importantly, as shown in Fig. 1g, the different colors in the lattice stripe images and the related fast Fourier transform (FFT)-filtered images are used to visualize the crystal defects in MnS_0.5_Se_0.5_@N-CNF, which are attributed to the increased disorder and distortion in the crystal structure caused by anion substitution of S with larger Se atoms. In comparison, the MnS@N-CNF exhibits a smaller interplanar spacing (0.213 nm) and fewer defects than MnS_0.5_Se_0.5_@N-CNF (Fig. 1h, i). The high-angle annular dark-field scanning TEM (HAADF-STEM) image of MnS_0.5_Se_0.5_@N-CNF and the corresponding energy-dispersive X-ray spectroscopy (EDX) elemental mappings reveal a homogeneous dispersion of MnS_0.5_Se_0.5_ nanocages within the carbon nanofibers (Fig. 1j). The EDX energy spectrum shows that the calculated atomic ratio of Mn:S:Se in MnS_0.5_Se_0.5_@N-CNF is approximately 1:0.5:0.5 (Fig. S3a, b). This ratio aligns with the elemental analysis results and corresponds to the stoichiometric ratio of MnS_0.5_Se_0.5_@N-CNF, confirming the successful introduction of Se species into the MnS_0.5_Se_0.5_@N-CNF lattice. Moreover, the morphologies of the MnS@N-CNF and MnSe@N-CNF composites, as revealed by SEM and TEM observations, show a similar network structure with carbon nanofibers interlaced and crossed in a staggered manner, while nanocubes uniformly arranged within the carbon nanofibers (Figs. S4 and S5).

**Fig. 2:**
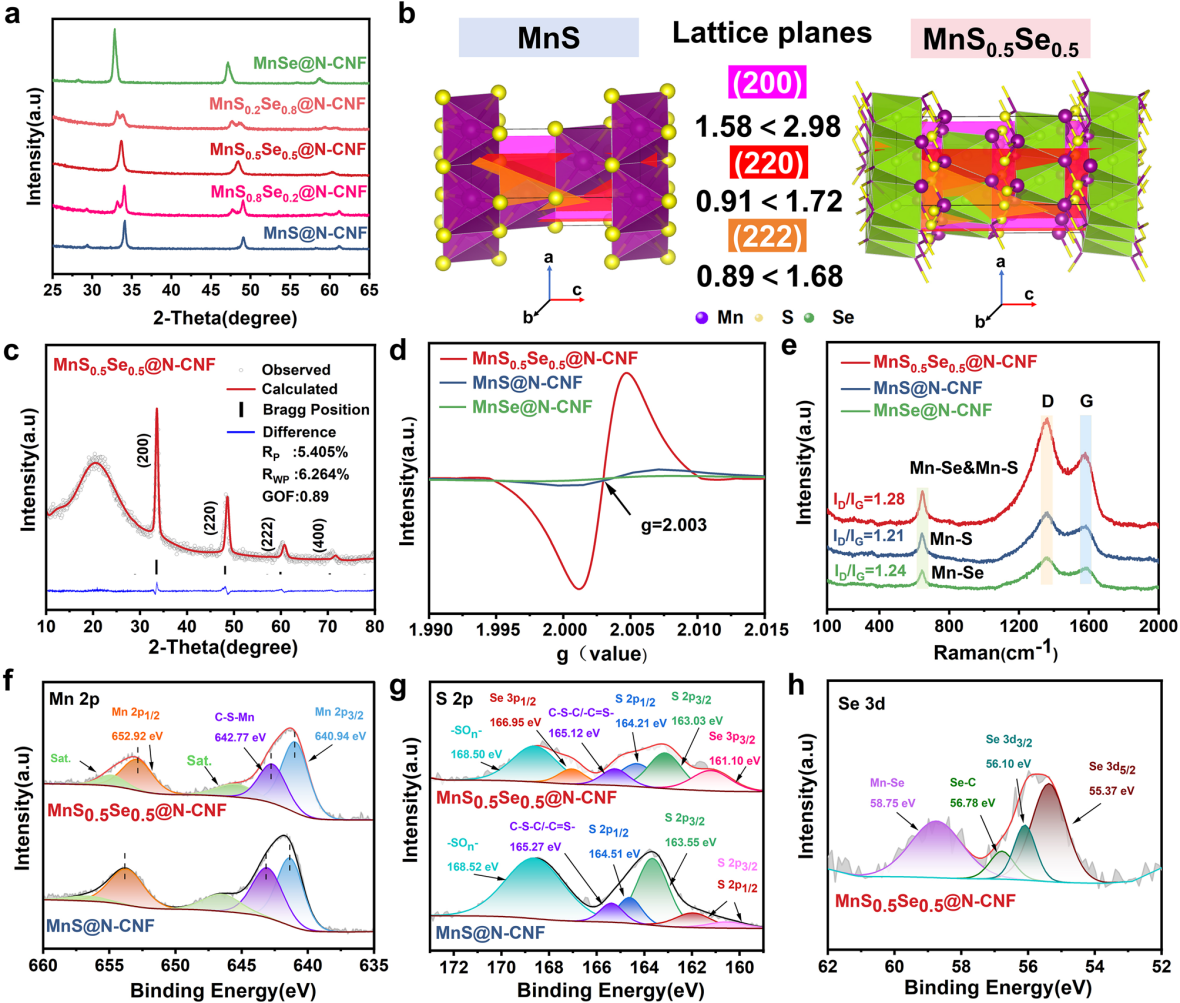
XRD patterns of **a** as-prepared Mn-based chalcogenide MnS_x_Se_1-x_@N-CNF. **b** Crystal structures of MnS_0.5_Se_0.5_ and MnS. **c** Rietveld-refined XRD result of the MnS_0.5_Se_0.5_@N-CNF sample. **d**, **e** Electron paramagnetic resonance results and Raman spectra for MnS_0.5_Se_0.5_@N-CNF, MnS@N-CNF and MnSe@N-CNF. **f** High-resolution XPS spectra of Mn 2*p*, and **g** high-resolution XPS spectra of S 2*p* for MnS_0.5_Se_0.5_@N-CNF and MnS@N-CNF. **h** High-resolution XPS spectrum of Se 3*d* in the MnS_0.5_Se_0.5_@N-CNF

The phase composition and crystal structure of the materials were identified through X-ray powder diffractometer (XRD) measurements. As shown in Fig. 2a, the main diffraction peaks from the sample MnS_0.5_Se_0.5_@N-CNF can belongs to the cubic crystal system with a Fm-3 m (225) space group (JCPDS No.01-089-4955) [33]. Notably, the (200), (220), and (222) planes of MnS_0.5_Se_0.5_@N-CNF present a significant shift to lower angles, indicating an expansion of the lattice spacing due to the incorporation of larger Se atoms into MnS at the atomic level, which is consistent with the TEM observations. To evaluate the impact of Se atoms on the crystal structure, we analyzed the structural characteristics using Rietveld-refined XRD. As can be seen from Figs. 2c and S6b, c, the XRD patterns of all samples are consistent with a cubic crystal system structure, resembling that of NaCl-type structure. In this structure, manganese ions occupy the positions of sodium-ions, while sulfur or selenium ions randomly replace the chloride ions. Figure 2b displays the three crystal planes (200), (220), and (222) of MnS_0.5_Se_0.5_ are larger than those of MnS, indicating that the incorporation of Se induces additional distortions in the crystal lattice, resulting in an expanded layer spacing that facilitates rapid Na^+^ diffusion. Moreover, the Mn-Se bond differs from the Mn-S bond due to the different electronegativity and bonding characteristics of Se, which alter the electronic structure of the material and cause charge redistribution within the MnS lattice [34]. This may lead to the formation of defects such as vacancies, interstitials, and antisite defects (where atoms occupy incorrect lattice positions), as confirmed by the electron paramagnetic resonance (EPR) spectrum (Fig. 2d) [35]. The symmetric Lorentzian line at a g-value of 2.003 for MnS_0.5_Se_0.5_@N-CNF, shows a stronger signal compared to MnS@N-CNF and MnSe@N-CNF, indicating a higher concentration of defects and more active sites.

Raman spectroscopy was also examined to verify the chemical composition and structure. As shown in Fig. 2e, the Raman spectra of MnS_0.5_Se_0.5_@N-CNF clearly reveal three characteristic peaks. The first peak, located in the Raman shift region of 580–700 cm^−1^, corresponds to the intrinsic vibrational modes of Mn-S and Mn-Se [36]. The intensity of this peak is higher than that of MnS@N-CNF and MnSe@N-CNF, indicating the successful complexation of S and Se with Mn, which is consistent with the XRD results. Two additional significant peaks, centered at ~ 1353 and ~ 1582 cm^−1^, correspond to typical features of *sp*^3^ hybridized disordered carbon (D-band) and *sp*^2^ hybridized graphitic carbon (G-band) [37]. The I_D_/I_G_ ratios of MnS_0.5_Se_0.5_@N-CNF, MnS@N-CNF and MnSe@N-CNF were calculated to be ≈1.28, 1.21, and 1.24, respectively. The higher I_D_/I_G_ ratio of MnS_0.5_Se_0.5_@N-CNF indicates that introducing Se atoms disrupts the symmetry of the carbon layer, resulting in a higher content of disordered carbon and an increased defect concentration within the carbon layer of the composite [38]. The weight percentage of carbon nanofiber (N-CNF) in the MnS_0.5_Se_0.5_@N-CNF composites was analyzed using thermogravimetric analysis (TGA) under air condition (Fig. S7). The exothermic peak in the differential thermogravimetry curve (red line in Fig. S7a) aligns with the weight loss curve, indicating chemical reactions and phase transitions. Based on the final residual weight, which was verified by the XRD pattern (Fig. S7b, c), the mass content of MnS_0.5_Se_0.5_ in MnS_0.5_Se_0.5_@N-CNF composite is calculated as about 88.9%, and the corresponding weight percentage of N-CNF in the sample is estimated to be 11.1%. The carbon nanofiber (N-CNF) content in MnS@N-CNF and MnSe@N-CNF composite materials are estimated to be approximately 31.5% and 42.3%, respectively. The specific area and pore structure of the samples were investigated by nitrogen adsorption/desorption measurements, as shown in Fig. S8a. All samples exhibit type-IV isotherms with a distinctive hysteresis loop in the relative pressure range of 0.46–0.94, suggesting the presence of micro-/mesoporous structures [39]. Furthermore, the MnS_0.5_Se_0.5_@N-CNF exhibits a greater specific Brunauer–Emmett–Teller (BET) surface area of 23.37 m^2^ g^−1^ in comparison with that of the MnS@N-CNF (12.28 m^2^ g^−1^) and MnSe@N-CNF (19.74 m^2^ g^−1^). The Barrett-Joyner-Halenda (BJH) pore size distribution plot (Fig. S8b) reveals that MnS_0.5_Se_0.5_@N-CNF has a wider variety of pore types, including microporous, mesoporous, and macroporous structures. The high porosity not only provides numerous channels for Na^+^ diffusion and storage within the electrode but also offers sufficient buffer space to accommodate the volume expansion of MnS_0.5_Se_0.5_@N-CNF during cycling.

X-ray photoelectron spectroscopy (XPS) was utilized to evaluate the surface electronic states and functional groups present in the synthesized samples. The XPS survey spectrum confirmed that the chemical composition of MnS_0.5_Se_0.5_@N-CNF includes elements such as Mn, S, Se, C, N, and O, as displayed in Fig. S9a, which is consistent with the results obtained from EDX mapping analysis. In the high-resolution C 1*s* spectra (Fig. S9d), distinct signals at binding energies of 284.5, 285.6, 286.6, and 288.1 eV correspond to C–C/C=C, C–N/C–O, C–N/C–S, and C=O groups, respectively [40]. The presence of N-doped species in the material is further confirmed by the high-resolution N 1*s* spectrum shown in Fig. S9g, which reveals four distinct nitrogen species: pyridinic N (398.4 eV), pyrrolic N (399.8 eV), graphitic N (400.8 eV), and oxygenated N (402.4 eV) [31]. Notably, the increase in graphitic N content with Se incorporation enhances both Na^+^ and electron transport capabilities in the MnS_0.5_Se_0.5_@N-CNF. The nitrogen content in the MnS_0.5_Se_0.5_@N-CNF composite is 12.2 at%, compared to 9.5 at% in the MnS@N-CNF composite. The high-resolution Mn 2*p* spectrum (Fig. 2f) reveals three peaks at 652.9, 642.7, and 640.9 eV. The peaks at 652.9 and 640.9 eV correspond to Mn 2*p*_1/2_ and Mn 2*p*_3/2_, respectively, indicating the presence of Mn^2+^. Additionally, the smaller peak at 642.7 eV likely represents the formation of C–S–Mn bonds between MnS and the carbon matrix [41]. Interestingly, the Mn 2*p* peaks of MnS_0.5_Se_0.5_@N-CNF shift to lower binding energies, indicating a reduction in the oxidation state of Mn due to bonding with the less electronegative Se atoms. This shift suggests that Se substitution tunes the d-band electronic structure of Mn atoms. In the S 2*p* high-resolution spectra (Fig. 2g), the spectra can be deconvoluted into four peaks. A pair of peaks at 163.03 and 164.21 eV corresponds to the S 2*p*_3/2_ and S 2*p*_1/2_ orbitals of Mn-S bond, while peaks at 161.10 and 166.95 eV are attributed to Se 3*p*_3/2_ and Se 3*p*_1/2_, respectively [6, 42]. Compared with that of MnS@N-CNF sample, both Mn 2*p* and S 2*p* for the MnS_0.5_Se_0.5_@N-CNF shift to lower binding energies, which is due to the incorporation of Se^2−^ into the MnS crystal lattice, resulting in a less electronegative chemical environment and lower binding energy of Mn-S. The peak at 165.12 eV is likely due to the formation of C–S bonds, possibly resulting from the interaction between sulfur and the carbon substrate, while the peak at 168.50 eV may be related to oxidized-S species formed upon exposure to air [43]. The high-resolution Se 3*d* spectra of the MnS_0.5_Se_0.5_@N-CNF in Fig. 2h present two typical peaks at the binding energies of 55.37 and 56.10 eV associating with the Se 3*d*_5/2_ and Se 3*d*_3/2_, respectively, characteristic of Se^2−^ (Mn–Se) [44]. Additionally, a peak at 56.78 eV indicates the presence of Se–C bonds, and the peak at 58.75 eV is attributed to SeO_x_, resulting from the oxidation of Se^2−^ in the air [45]. These results provide insights into the chemical bonding and electron transfer in the C–Mn/C–S/C–Se architecture of MnS_0.5_Se_0.5_@N-CNF, which not only reduces the electron polarization but also accelerate the Na^+^ and electron transfer.

### Test and Analysis of Electrochemical Properties of the Samples

The electrochemical performance of the prepared composite anodes for sodium-ion storage was initially evaluated using half-coin cells with metallic sodium foil as the counter/reference electrode. The typical cyclic voltammetry (CV) curves of MnS_0.5_Se_0.5_@N-CNF for the initial three cycles, measured at a scan rate of 0.1 mV s^−1^ within the voltage window of 0.01–3.00 V, are shown in Fig. S10. During the first cathodic scan, two relatively weak peaks appeared at approximately 1.58 and 2.08 V, which can be attributed to the multi-step intercalation of Na^+^ into the interlayers of MnS_0.5_Se_0.5_@N-CNF without phase transition [6]. Subsequently, two sharp reduction peaks at around 0.60 and 0.10 V are likely due to the conversion reaction, resulting in the formation of metallic Mn and Na-S/Na-Se compounds, accompanied by the formation of an irreversible solid electrolyte interface (SEI) film [46]. In the subsequent anodic scan, the oxidation peaks at approximately 1.54, 2.02, and 2.10 V correspond to the stepwise desodiation reactions [47]. In the subsequent anodic scan, the oxidation peaks at approximately 1.54, 2.02, and 2.10 V correspond to the stepwise desodiation reactions. The MnS_0.5_Se_0.5_@N-CNF electrodes show higher peak currents compared to MnS@N-CNF and MnSe@N-CNF, further demonstrating that the introduction of Se enhances the electrochemical reaction activity. In the subsequent cycles, the MnS_0.5_Se_0.5_@N-CNF electrodes displayed nearly overlapping redox peaks, indicating improved stability and reversibility of the Na^+^ storage reaction process after Se doping.

**Fig. 3:**
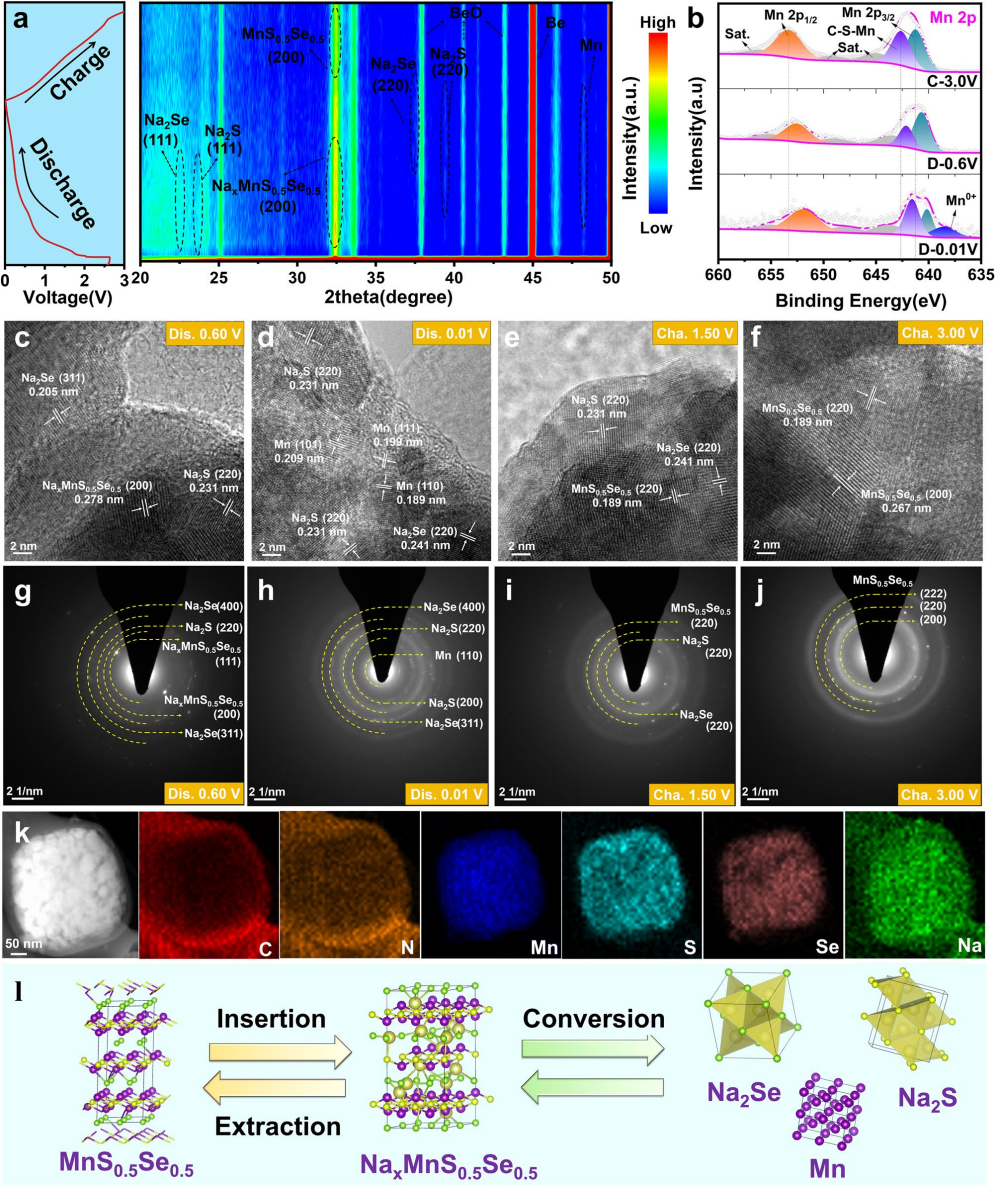
**a** In situ XRD with a 2D contour plot of the MnS_0.5_Se_0.5_@N-CNF electrode during the initial cycle. **b** High-resolution XPS Mn 2*p* spectra of the MnS_0.5_Se_0.5_@N-CNF electrode at different initial discharged and charged states. Ex-situ HRTEM images and the corresponding selected-area electron diffraction patterns of the MnS_0.5_Se_0.5_@N-CNF electrode at different initial discharged and charged states: **c**, **g** discharged state of 0.60 V, **d**, **h** discharged state of 0.01 V, **e**, **i** charged state of 1.50 V, **d**, **h** charged state of 3.00 V, and **k** EDS elemental mappings of discharging to 0.01 V. **l** The corresponding schematic illustration of Na^+^ storage mechanism for the MnS_0.5_Se_0.5_@N-CNF composite

Furthermore, in-situ XRD, ex-situ XPS, and ex-situ HRTEM were employed to characterize the structural transformation and phase evolution of MnS_0.5_Se_0.5_@N-CNF. Figure 3a shows the in-situ XRD patterns of MnS_0.5_Se_0.5_@N-CNF for the sodiation/desodiation processes. The peak at around 38.5°, 41.2°, 43.9°, and 45.8° belongs to the Be and BeO, which are attributed to the in-situ cell window [26]. The contour plot reveals prominent diffraction peaks at 33.5° in the pristine MnS_0.5_Se_0.5_@N-CNF, corresponding to the (200) plane. As the discharge process to 1.5 V, the characteristic peaks shifted to lower angles, indicating the insertion of Na^+^ into MnS_0.5_Se_0.5_, forming an intermediate Na_x_MnS_0.5_Se_0.5_. When the electrode is further discharged to 0.60 V, the distinct peaks from Na_x_MnS_0.5_Se_0.5_ gradually increase in intensity. Meanwhile, new peaks appear at ≈22.6° and 37.3°, corresponding to the (111) and (220) planes of Na_2_Se (JCPDS No. 04-003-6921), and at ≈23.5° and 38.9°, corresponding to the (111) and (220) planes of Na_2_S (JCPDS No. 01-071-4842). When the electrode is fully discharged to 0.01 V, the Na_2_Se and Na_2_S signal still remains, and weak characteristic diffraction peaks appear at 48.0°, corresponding to metallic Mn (JCPDS No. 99-000-2278). The intensity of the Na_x_MnS_0.5_Se_0.5_ peaks gradually weakens, indicating the conversion reaction between Na_x_MnS_0.5_Se_0.5_ and Na^+^, forming metallic Mn, Na_2_Se, and Na_2_S. During the charging process, the Na_2_S and Na_2_Se peaks gradually weaken and almost disappear by the end of the charge (3.00 V), suggesting that the conversion reaction between Na_2_S, Na_2_Se and Mn allows the original MnS_0.5_Se_0.5_ phase to fully recover, which is consistent with other reported manganese-based compound electrodes for SIBs [9, 16].

Subsequently, ex-situ XPS was employed to further analyze the changes in the valence state of MnS_0.5_Se_0.5_@N-CNF during cycling. As shown in Fig. 3b, the high-resolution Mn 2*p* spectrum reveals that when the electrode is discharged from 0.60 to 0.01 V, the Mn 2*p* peak shifts significantly toward lower binding energy. Notably, a clear characteristic peak of Mn^0^ 2*p* is observed, which may result from the reaction between Na^+^ and MnS_0.5_Se_0.5_ [48]. Upon charging the electrode back to 3.00 V, the Mn^0^ peak disappears completely, and the Mn 2*p* peak subtly shifts toward higher binding energy, closely matching the pristine state. A similar trend is observed in the high-resolution spectra of S 2*p* and Se 3*d* (Fig. S12a, b), where the energy shift toward lower binding energies from 0.60 V to the fully discharged state and the corresponding intensity changes are even more pronounced. These results indicate strong chemical bonding between Na^+^ and heteroatom-doped active sites, suggesting the formation of Na_2_S and Na_2_Se [6]. Upon reverse charging to 3.00 V, the peak intensities of S and Se increase, and their binding energies shift back to higher values, confirming that the electrochemical reaction exhibits good reversibility.

The physical phase transformation of MnS_0.5_Se_0.5_@N-CNF was further verified by ex-situ HRTEM characterization. As depicted in Fig. 3c, d, after discharging the pristine MnS_0.5_Se_0.5_ phase to 0.60 V, the lattice fringes of the (200) crystal plane expand slightly to 0.278 nm due to Na^+^ uptake. Additionally, interplanar spacings of 0.205 and 0.231 nm are observed, corresponding to the (311) crystal plane of Na_2_Se and the (220) plane of Na_2_S, respectively [27]. Upon further discharge to 0.01 V, the interplanar spacings of the final product are 0.231, 0.241, 0.209, 0.199, and 0.189 nm, corresponding to the (220) plane of Na_2_S, the (220) plane of Na_2_Se, and the (101), (111), and (110) planes of metallic Mn, respectively [6]. The corresponding SAED pattern displays a series of diffraction rings (Fig. 3g, h), corresponding to the cubic Mn (JCPDS No. 99-000-2278), cubic Na_2_S (JCPDS No. 01-071-4842), and Na_2_Se (JCPDS No. 04-003-6921), confirming the conversion processes. The EDS element mapping from STEM images shown in Fig. 3k further confirms that the carbon nanofiber-encapsulated porous nanocube structure is well maintained, with Mn, S, Se, and Na uniformly distributed within the C matrix, demonstrating high structural stability. Upon charging to 1.50 V, the SAED pattern (Fig. 3i) and HRTEM result (Fig. 3e) show the Na_2_S/Na_2_Se underwent partial transformation into MnS_0.5_Se_0.5_, with some Na_2_S and Na_2_Se remaining. Simultaneously, the lattice fringe of Mn disappears, demonstrating the Na-ion extraction process. Upon charging to 3.00 V, the diffraction rings and lattice fringes of Na_2_S and Na_2_Se disappear, replaced by those of the MnS_0.5_Se_0.5_, indicating the highly reversible conversion reaction (Fig. 3f, j). From the above results, the MnS_0.5_Se_0.5_@N-CNF exhibits a stepwise electrochemical process, with synergistic effects between the conversion and Na-ion (de)intercalation reactions, as visually represented in Fig. 3l. Overall, the detailed working processes of MnS_0.5_Se_0.5_@N-CNF is summarized as follows:

Sodiation Process:

(i) MnS_0.5_Se_0.5_ + xNa^+^ + xe^−^ → Na_*x*_MnS_0.5_Se_0.5_
(ii) Na_*x*_MnS_0.5_Se_0.5_ + (2-x) Na^+^  + (2−x) e^−^ → Mn + 0.5Na_2_S + 0.5Na_2_Se

Desodiation Process:

(iii) Mn + 0.5Na_2_S + 0.5Na_2_Se → MnS_0.5_Se_0.5_ + 2Na^+^ + 2e^−^

**Fig. 4:**
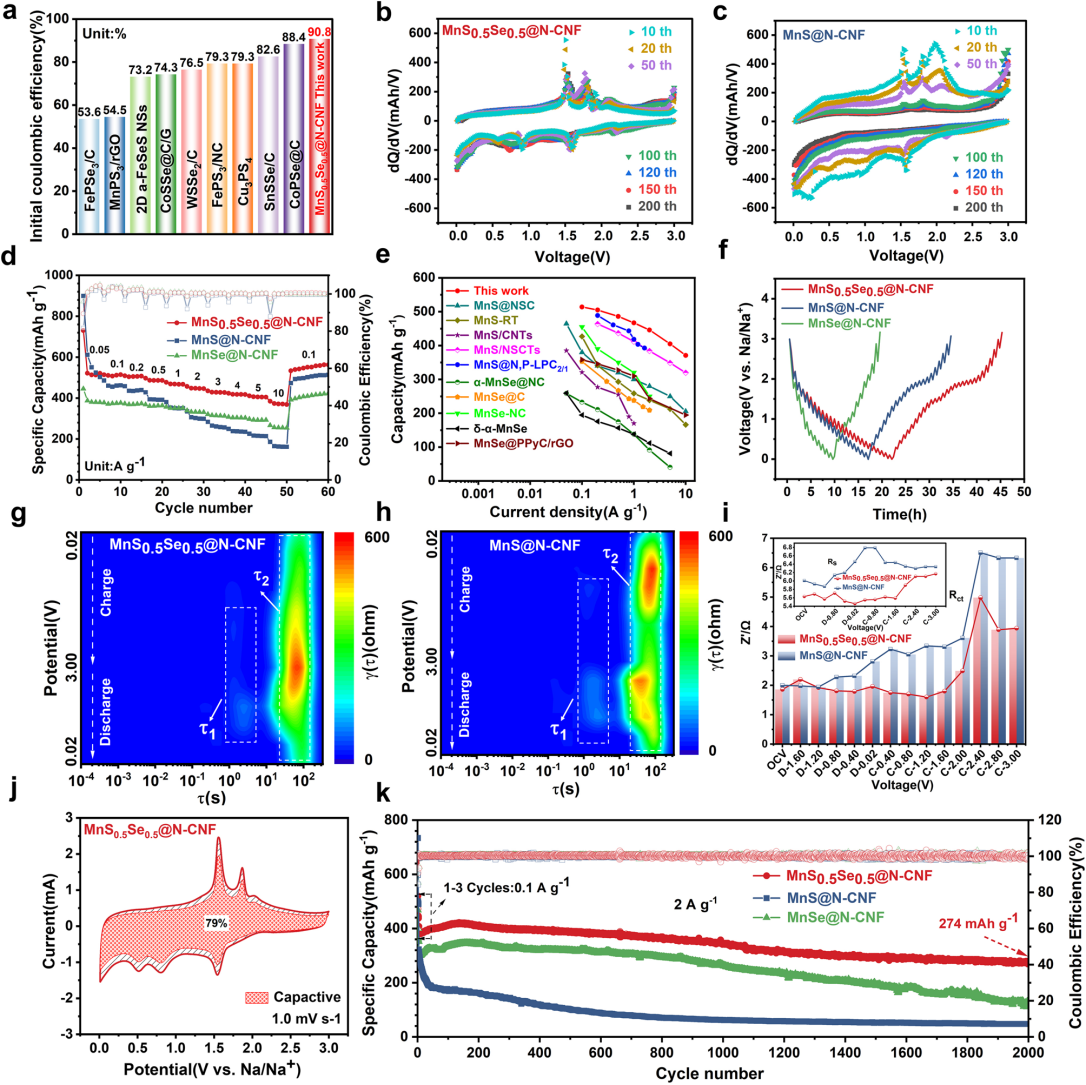
**a** Comparison of ICE at 0.1 A g^−1^,** b** differential charge versus voltage curve of MnS_0.5_Se_0.5_@N-CNF and **c** MnS@N-CNF electrodes. **d** Rate performance of MnS_0.5_Se_0.5_@N-CNF, MnS@N-CNF and MnSe@N-CNF electrodes. **e** Comparison of rate performance of MnS_0.5_Se_0.5_@N-CNF with the reported MnSe and MnS-based anodes for SIBs. **f** GITT curves of MnS_0.5_Se_0.5_@N-CNF, MnS@N-CNF and MnSe@N-CNF electrodes. DRT contour plots from EIS measurements of **g** MnS_0.5_Se_0.5_@N-CNF and **h** MnS@N-CNF at different potentials.** i** The evolution of R_SEI_ and R_ct_ during cycling in MnS_0.5_Se_0.5_@N-CNF and MnS@N-CNF electrodes. **j** Capacitive contribution at 1.0 mV s^−1^ of the MnS_0.5_Se_0.5_@N-CNF electrode. **k** Long-term cycling performance of the MnS_0.5_Se_0.5_@N-CNF, MnS@N-CNF and MnSe@N-CNF electrodes at 2.0 A g^−1^

Due to its well-designed nanostructure, excellent sodium storage reversibility, and high defect concentration, the carbon nanofiber-encapsulated porous nanocube MnS_0.5_Se_0.5_@N-CNF nanocomposite is expected to exhibit outstanding performance in SIBs. Figure S13a-c shows the galvanostatic charge–discharge (GCD) curves of each sample for the initial three cycles at 0.1 A g^−1^, where the corresponding voltage platforms align well with the redox behaviors observed in the CV results. The MnS_0.5_Se_0.5_@N-CNF electrode delivers an initial discharge and charge capacities of 613.2/556.8 mAh g^−1^, corresponding to a high initial Coulombic efficiency (ICE) of 90.8%, which is higher than that of MnS@N-CNF (682.2/553.0 mAh g^−1^, 81.1%). Such high ICE of MnS_0.5_Se_0.5_@N-CNF exceeds the values reported for other metal dichalcogenides with multiple anions (Fig. 4a) [49–57]. The improvement in the ICE of the MnS_0.5_Se_0.5_@N-CNF electrode in SIBs can be attributed to the following factors: 1) the substitution of S by Se causes lattice distortion, which expands the ion channels, facilitating Na⁺ diffusion, 2) the incorporation of carbon nanofibers improves electrical conductivity while also providing mechanical support, maintaining the structural integrity, 3) the strong interfacial interaction between MnS_0.5_Se_0.5_ and the carbon nanofibers contributes to the formation of a stable SEI film. These aspects promote the reversible reaction of sodium-ions, reduce irreversible losses and capacity degradation in multiple ways. Moreover, the capacity of MnS_0.5_Se_0.5_@N-CNF electrode gradually increases after the initial cycles and begins to stabilize after 100 cycles, demonstrating a relatively high and stable reversible capacity of 595.1 mAh g^−1^ after 200 cycles (Fig. S14). This increase is likely due to the pulverization of MnS_0.5_Se_0.5_ nanoparticles, which creates more active sites for Na^+^ storage. In contrast, the MnSe@N-CNF electrode exhibits a similar phenomenon, with its capacity sharply decaying after 100 cycles before stabilizing at 343.6 mAh g^−1^ after 200 cycles. The capacity of MnS@N-CNF, however, continuously decays from 537.0 to 181.0 mAh g^−1^ after 200 cycles. Moreover, the morphology of MnS_0.5_Se_0.5_@N-CNF still maintains its original structure after a long cycle of charge and discharge (Fig. S15), indicating that it has excellent structural stability. Charge–voltage curves collected at different cycles further confirm the stable and reversible Na^+^ reactions of the MnS_0.5_Se_0.5_@N-CNF electrode (Fig. 4b). However, for the MnS@N-CNF electrode, the peaks around 1.5–2.2 V weaken and eventually disappear with increasing cycles, corresponding to the conversion reaction of MnS. This indicates that during electrode pulverization, the shuttle effect of polysulfide intermediates becomes severe, leading to poor structural integrity (Fig. 4c). Furthermore, the MnS_0.5_Se_0.5_@N-CNF electrode delivered superior rate performance (Fig. 4d). The capacities for MnS_0.5_Se_0.5_@N-CNF are 520.9, 514.5, 507.7, 486.5, 467.2, 446.8, 427.8, 418.3, and 405.2 mAh g^−1^ at 0.05, 0.1, 0.2, 0.5, 1, 2, 3, 4, and 5 A g^−1^, respectively. Notably, it achieves a high reversible capacity of 370.5 mAh g^−1^ even at a high current density of 10 A g^−1^. When the current density is restored to 0.1 A g^−1^, a high average reversible capacity of 539.5 mAh g^−1^ can be maintained, and the capacity continues to grow, demonstrating rapid redox reactions and low polarization. In contrast, under the same testing conditions, MnS@N-CNF electrodes exhibit rapid capacity decay from 524.5 to 161.9 mAh g^−1^. The rate performance of MnS_0.5_Se_0.5_@N-CNF is significantly better than that of MnS@N-CNF and MnSe@N-CNF, standing out among other reported MnSe and MnS-based anodes for SIBs (Fig. 4e) [7, 36, 40, 58–64]. In addition, the discharge/charge voltage curves are stable, with the electrode polarization increasing slowly (Fig. S16a-c), further confirming the strong stability and excellent Na^+^ capturing ability of MnS_0.5_Se_0.5_@N-CNF as the current density increases, which is crucial for high-power battery applications. Moreover, the MnS_0.5_Se_0.5_@N-CNF electrode exhibited outstanding rate performance even under high mass loading, as shown in Fig. S17. The areal capacity increases nearly linearly with current density from 0.1 to 5 A g^−1^ when the mass loading is up to 2.9 mg cm^−2^. However, at a higher mass loading of 4.1 mg cm^−2^, the areal capacity deviates from linearity and begins to plateau. This phenomenon can be attributed to charge transport limitations within the electrolyte at such high mass loading, where the penetration depth of the ionic current becomes insufficient, leading to reduced utilization of active materials. Nevertheless, across different mass loadings, the capacity of the MnS_0.5_Se_0.5_@N-CNF electrode consistently returns to its initial state when the current density is restored to 0.1 A g^−1^, demonstrating its excellent reversibility.

### Kinetic Analysis of the Samples

The galvanostatic intermittent titration technique (GITT) was used to in-situ evaluate the interfacial reaction resistance of as-prepared electrodes during the sodiation and desodiation processes. The MnS_0.5_Se_0.5_@N-CNF electrode exhibits smaller overpotentials and higher Na^+^ diffusion coefficients (D_Na+_) during both the Na⁺ insertion and extraction processes compared to the MnS@N-CNF and MnSe@N-CNF electrode (Fig. S18), indicating improved Na^+^ diffusion kinetics. This is further corroborated by in-situ electrochemical impedance spectroscopy (EIS) analyses, which reveal the impact of Se doping on charge transport kinetics. As shown in Fig. S19a, d, the EIS profiles of the MnS_0.5_Se_0.5_@N-CNF and MnS@N-CNF electrodes are similar, featuring a semicircle in the high-frequency region, corresponding to charge transfer resistance (R_ct_) and SEI film resistance (R_s_), and an oblique line in the low-frequency region corresponding to Warburg impedance (Z_w_), which represents the diffusion of Na^+^ ions [65]. To clarify this disparity and separate the overlapping electrochemical phenomena existing in conventional Nyquist plots, the distribution of relaxation time (DRT) analysis was adopted for quantitative deconvolution (Fig. S19b, c and e, f), and the corresponding contour plots were presented in Fig. 4g, h. The τ₁ peak (about 10^0^–7.5 × 10^0^ s) corresponds to the migration of Na^+^ across the surface of the porous network. Due to the carbon nanofiber structure, MnS@N-CNF and MnS_0.5_Se_0.5_@N-CNF exhibit minimal impedance contributions during this process. The τ₂ peak (about 10^1^–2 × 10^2^ s) is associated with the ion diffusion impedance in the low-frequency region of the EIS, reflecting the deep diffusion of Na^+^ into the bulk electrode. Compared with MnS@N-CNF, MnS_0.5_Se_0.5_@N-CNF has a larger specific surface area, which shortens the ion transport channels. Moreover, the introduction of Se improves the internal electronic structure of the material and accelerates the ion mobility, thus alleviating this diffusion limitation. The EIS curves of electrodes were fitted using an equivalent circuit (Fig. S20) [66]. Compared with MnS@N-CNF, the MnS_0.5_Se_0.5_@N-CNF electrode shows ultralow and highly stable charge transfer resistances across different (de)sodiation states (Fig. 4i), indicating the ultra-fast and robust reaction kinetics during continuous structural evolution. A kinetic study based on cyclic voltammetry (CV) curves at various scan rates was conducted to further understand the mechanism behind the rapid Na⁺ ion storage in the MnS_0.5_Se_0.5_@N-CNF electrode. As depicted in Fig. S22a-c, compared with the CV curves of MnS@N-CNF and MnSe@N-CNF electrode, the MnS_0.5_Se_0.5_@N-CNF maintains a consistent CV shape as the scan rate increases from 0.2 to 1.2 mV s^−1^, demonstrating minimal electrochemical polarization and excellent electrochemical reversibility during the charging/discharging process. The redox peak b value of the MnS_0.5_Se_0.5_@N-CNF electrode ranges from 0.51 to 1.37, with both cathodic and anodic peak currents higher than those of the MnS@N-CNF electrode (Fig. S22d-f), indicating that the electrode is primarily surface capacitance-controlled in its Na⁺ storage mechanism. As shown in Fig. 4j, the MnS_0.5_Se_0.5_@N-CNF electrode makes a significant contribution to capacitance of 79.0% at 1.0 mV s^−1^, which is higher than 61.0% of the MnS@N-CNF electrode (Fig. S23a). Moreover, when the scan rate increases from 0.2 to 1.2 mV s^−1^, the capacitive contribution of the MnS_0.5_Se_0.5_@N-CNF electrode increases from 67.2% to 84.5%, which is significantly higher than that of the MnS@N-CNF electrode (Fig. S23b). These results indicate that the implantation of anion Se defects into metal sulfides is more conducive to surface-induced pseudocapacitive charge storage. The outstanding electrochemical kinetics of MnS_0.5_Se_0.5_@N-CNF is a key factor behind its excellent cycling stability, with a capacity of 274.0 mAh g^−1^ after 2000 cycles at 2 A g^−1^, retaining 68.5% of its capacity retention. The capacity of the MnS_0.5_Se_0.5_@N-CNF electrode gradually increases after the initial cycles, which can be attributed to the continuous activation of active materials and the decomposition of electrolytes during cycling. In contrast, the MnS@N-CNF anode displays a much poorer cycling stability, with only 26.3% capacity retention after 2000 cycles (Fig. 4k). Moreover, the MnS_0.5_Se_0.5_@N-CNF anode maintains stable discharge/charge voltage profiles with minimal electrode polarization over prolonged cycling. In comparison, the MnS@N-CNF anode suffers from limited electrical conductivity, necessitating higher operating voltages to sustain performance. This leads to increased polarization, lower capacity output, and accelerated capacity degradation. Furthermore, the MnS_0.5_Se_0.5_@N-CNF anode also demonstrates highly stable cycling performance at 5 A g^−1^, retaining 190.8 mAh g^−1^ after over 5000 cycles with a capacity retention of 65.5% and nearly 100% Coulombic efficiency (Fig. S24). The excellent sodium storage performance of MnS_0.5_Se_0.5_@N-CNF is primarily attributed to its porous structure and nanofiber carbon framework, which mitigate volume expansion and maintain high structural integrity during Na^+^ insertion and extraction. Additionally, the interfacial effect resulting from the incorporation of S and Se into the transition metal dichalcogenide promotes the transfer of interfacial electrons. Consequently, the MnS_0.5_Se_0.5_@N-CNF electrode is expected to be a high-capacity and durable anode material for SIBs.

### DFT Analysis of the Influence of Se Doping on Na^+^ Storage

**Fig. 5:**
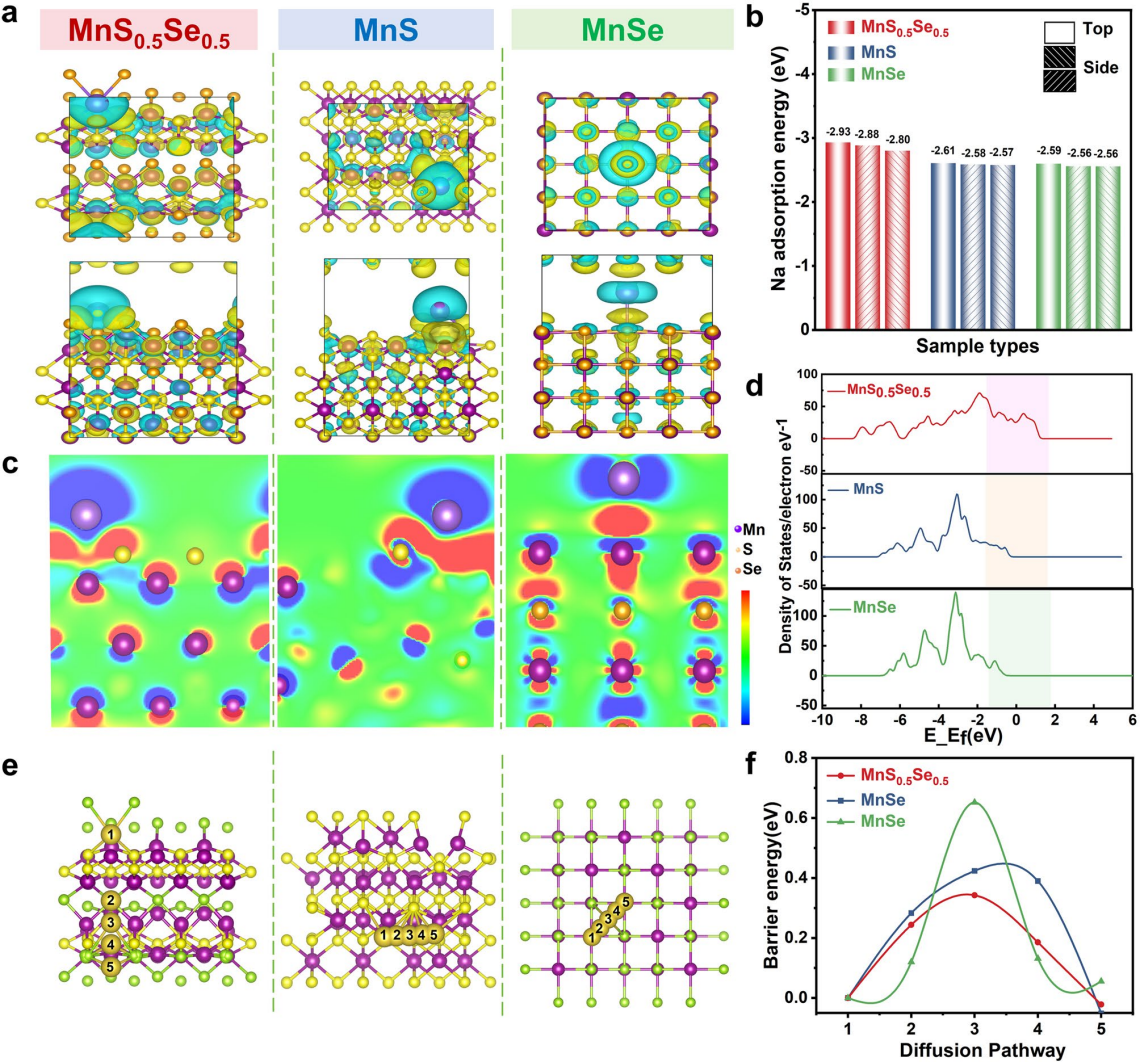
**a** Na-ion adsorption energy at different active sites of MnS_0.5_Se_0.5_, MnS and MnSe. **b** Na-ion adsorption energy at different active sites of MnS_0.5_Se_0.5_, MnS and MnSe. **c** 2D projection of charge density contour of MnS_0.5_Se_0.5_, MnS and MnSe. **d** Density of states (DOS) of MnS_0.5_Se_0.5_, MnS and MnSe. **e** Illustration of the Na-ion diffusion path in MnS_0.5_Se_0.5_, MnS and MnSe. **f** Na^+^ diffusion energy barriers of MnS_0.5_Se_0.5_, MnS and MnSe

To gain a deeper understanding of the significantly enhanced electrochemical activity and stability of MnS_0.5_Se_0.5_@N-CNF, we performed density functional theory (DFT) calculations to explore its structural advantages. First-principles calculations were conducted to investigate the impact of S/Se doping on Mn-based materials for Na^+^ storage. The adsorption energy (ΔEa) of Na^+^ on the activity sites in all configurations were calculated. As shown in Fig. 5b, MnSe (− 2.59 eV) and MnS (− 2.61 eV) exhibited a poor adsorption capacity for Na^+^ (Figs. S26 and S27). However, with the introduction of Se and a few S atoms into the Mn-based materials, the Na^+^ adsorption on the site was significantly enhanced, affording a ΔEa of MnS_0.5_Se_0.5_ (− 2.93 eV) (Fig. S25). This improvement in Na⁺ adsorption can be attributed to the bonding between Se and S atoms, as well as the larger radius of the Se atom, which creates protruding structures on the Mn-based materials, increasing the interlayer spacing and boosting the sodium-ion energy storage capacity. To obtain a deeper perspective of the doping effects, the total DOS (TDOS) were essentially taken a deep perspective (Fig. 5d). The MnS_0.5_Se_0.5_ exhibited a higher DOS peak near the Fermi level after Se doping, indicating that Se facilitated the electrical conductivity of the materials. Furthermore, the MnS_0.5_Se_0.5_ shows higher reactivity of the active sites than others according to the associated the local Fermi softness analysis. Additionally, the charge density difference was examined to understand the bonding properties of the adsorbed Na⁺ in the corresponding model (Fig. 5a). In the 2D projection of the charge density contour, MnS_0.5_Se_0.5_, MnS and MnSe were compared (Fig. 5c). The red and blue regions indicate higher and lower reactivity, respectively. The MnS_0.5_Se_0.5_ exhibited significantly more red areas, favoring more effective energy storage compared to the other two samples. To further investigate the fast kinetics of MnS_0.5_Se_0.5_, MnS and MnSe, the diffusion barrier energies and the minimum energy paths for Na^+^ were calculated (Fig. 5e, f). MnS_0.5_Se_0.5_ exhibited the lowest diffusion energy barrier (0.34 eV) compared to MnS (0.42 eV) and MnSe (0.65 eV). This suggests that the Se and N dopants contribute to improved diffusion kinetics and reduced diffusion resistance for Na^+^. In conclusion, the DFT results indicate that the excellent performance of MnS_0.5_Se_0.5_ can be attributed to the large number of defects, additional active sites, and the expansion of layer spacing caused by Se/S doping. These factors enhance the Na^+^ adsorption capacity, leading to rapid electrochemical reaction kinetics and improved capacity performance for the anode.

### Electrochemical Performance Tests in Sodium-Ion Full Cell

**Fig. 6:**
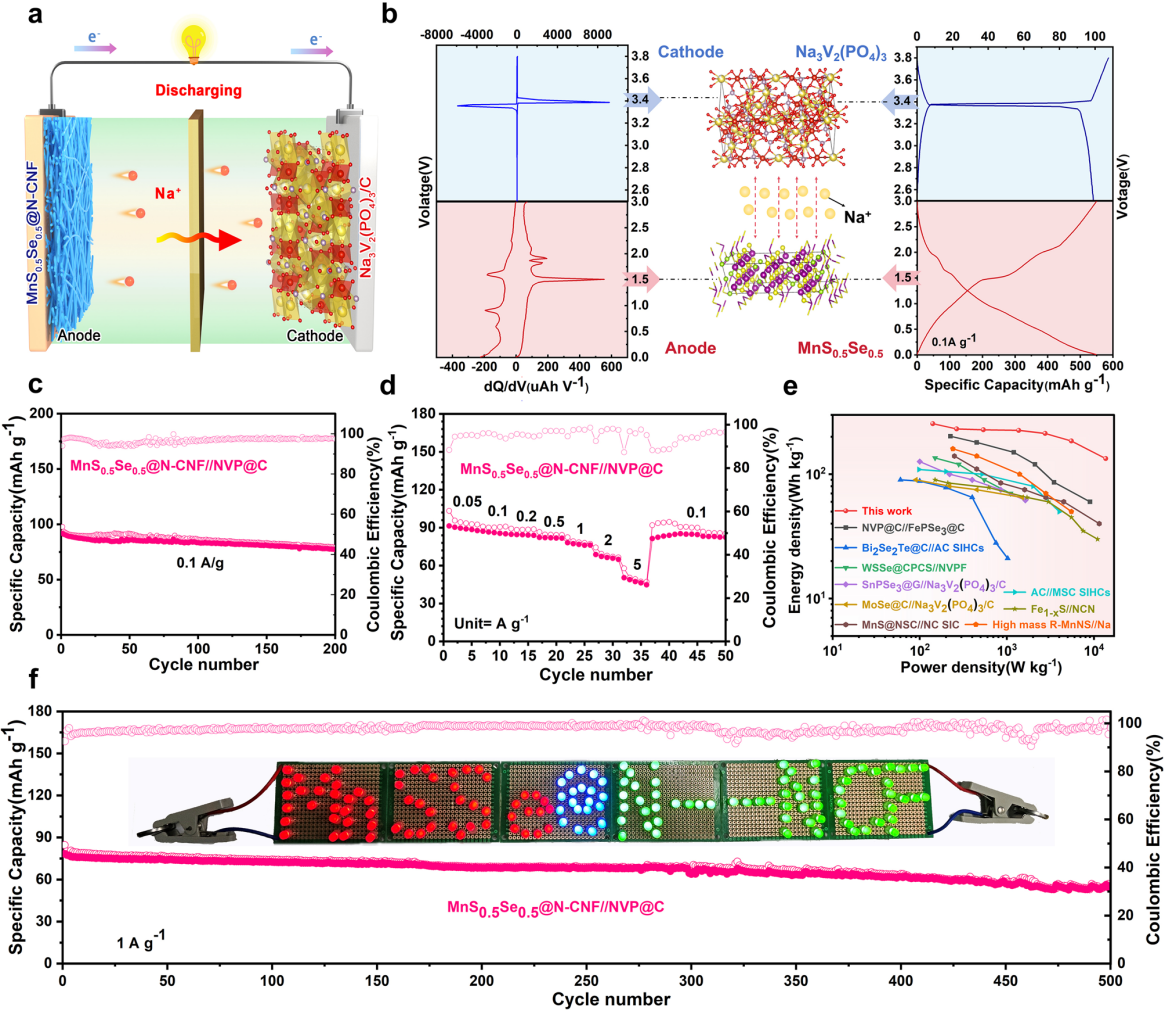
**a** Schematic illustration of the sodium-ion full battery with MnS_0.5_Se_0.5_@N-CNF //NVP@C couple. **b** Electrochemical performances of the as-prepared full cell based on the MnS_0.5_Se_0.5_@N-CNF anode and NVP@C cathode: left) charge versus voltage curves and right) charge–discharge profiles of the corresponding half-cells. **c** Cycling stability at 0.1 A g^−1^, and **d** rate performance of the full battery. **e** Comparison of the energy/power density of the MnS_0.5_Se_0.5_@N-CNF //NVP@C device with previously reported SIBs full battery. **f** Long-term cycle performance of the full cell at 1 A g^−1^ (Inset of photograph of different colors of LED lights powered by the full battery)

Additionally, a sodium-ion full cell was assembled via employing the MnS_0.5_Se_0.5_@N-CNF as anode and Na_3_V_2_(PO_4_)_3_@C (NVP@C) as cathode to further evaluate the application potential of SIBs device, as illustrated in the schematic diagram in Fig. 6a. The electrochemical properties and morphological characterizations of NVP@C are detailed in Fig. S28. Figure 6b shows the charge/discharge curves of the NVP@C cathode and MnS_0.5_Se_0.5_@N-CNF anode. The full cell exhibits a voltage difference between the cathode and anode. The integral calculation results in an operating voltage of 1.90 V during discharge. The charge/discharge curves for the first three cycles demonstrate excellent overlap, achieving a coulombic efficiency close to 100% (Fig. S29). The cycling performance is further illustrated in Fig. 6c, revealing a remaining reversible capacity of 77 mAh g^−1^ after 200 cycles, with a capacity retention of 85.6%. The capacity of the full battery is obtained based on the total mass of the positive and negative electrode materials. Moreover, the full cell demonstrates superior rate performance, with discharge capacities of 90.3, 87.2, 84.5, 82.1, 77.3, 67.2, and 48.9 mAh g^−1^ at the current densities of 0.05, 0.1, 0.2, 0.5, 1, 2, and 5 A g^−1^, respectively (Fig. 6d). When the current density is reduced back to 0.1 A g^−1^, a reversible capacity of 81.4 mAh g^−1^ can be recovered. The charge/discharge curves at different current densities show minimal polarization, even at the high current density of 5 A g^−1^ (Fig. S30), verifying splendid rate ability. The calculated energy and power densities of MnS_0.5_Se_0.5_@N-CNF//NVP@C full cell are presented in Fig. 6e. The full cell delivers a maximum energy density of 254 Wh kg^−1^ at a power density of 141 Wkg^−1^. Even at the high-power density of 13,650 W kg^−1^, the energy density remains at 133.5 Wh kg^−1^. This performance is comparable to, or even surpasses, that of some representative sodium-ion full cells reported in the literature [7, 19, 21, 67–72]. Moreover, the MnS_0.5_Se_0.5_@N-CNF//NVP@C full cell exhibits long cycle performance at 1.0 A g^−1^ (Fig. 6f), with a reversible capacity of 54 mAh g^−1^ after 500 cycles, demonstrating excellent durability. Additionally, a series of light-emitting diodes (LEDs) are successfully illuminated using two MnS_0.5_Se_0.5_@N-CNF//NVP@C button cells (inset in Fig. 6f), further highlighting the potential for practical applications in the future.

## Conclusions

In conclusion, we demonstrated the fabrication of necklace-like MnS_0.5_Se_0.5_@N-CNF using a simple electrospinning method. The porous and necklace-like nanofiber network structure provides an efficient ion transfer pathway and alleviates stress during the charge–discharge cycles. Comprehensive experimental investigations and DFT calculations indicate that the incorporation of Se not only helps to increase the active surface area and enhances the electrical conductivity, but also improves the adsorption capacity and decreases the migration energy barrier of Na^+^, thus accelerating the electrochemical reaction kinetics and strengthening the cycling stability. Additionally, the structural evolution mechanism of MnS_0.5_Se_0.5_@N-CNF during the sodiation/desodiation process was studied using in-situ XRD, ex-situ TEM and XPS. Thanks to its unique hierarchical structure and the synergistic combination of components, the MnS_0.5_Se_0.5_@N-CNF electrode demonstrates a high specific capacity, outstanding rate performance (370.5 mAh g^−1^ at 10 A g^−1^), and remarkable long-term cycling performance over 5000 cycles. Furthermore, when paired with Na_3_V_2_(PO_4_)_3_@C to form a MnS_0.5_Se_0.5_@N-CNF//NVP@C sodium-ion battery, the energy density reaches as high as 254 Wh kg^−1^. This research effectively addresses the challenges associated with TMSs anode for SIBs and provides new insights for the development of advanced battery materials.

## Supplementary Information

Below is the link to the electronic supplementary material.

Supplementary file1 (DOCX 8300 kb)
