# Supplementary material for: Se-Regulated MnS Porous Nanocubes Encapsulated in Carbon Nanofibers as High-Performance Anode for Sodium-Ion Batteries
Source: Nanomicro Lett. 2025 Apr 28;17:237. doi: 10.1007/s40820-025-01767-4 (PMC12037962; doi:10.1007/s40820-025-01767-4)
Supplement: Supplementary file 1 — Supplementary file1 (DOCX 8300 kb) [file 40820_2025_1767_MOESM1_ESM.docx]

Supporting Information for

**Se-Regulated MnS Porous** **Nanocubes Encapsulated in Carbon Nanofibers as High-Performance Anode for Sodium-Ion Batteries**

Puwu Liang^1,2^, Duo Pan^1^, Xiang Hu^1,^* , Ke R. Yang^5^, Yangjie Liu^1^, Zijing Huo^1,2^, Zheng Bo^3^, Lihong Xu^1^, Junhua Xu^4,^*, Zhenhai Wen^1,^*

^1^ State Key Laboratory of Structural Chemistry, and Fujian Provincial Key Laboratory of Materials and Techniques toward Hydrogen Energy, Fujian Institute of Research on the Structure of Matter, Chinese Academy of Sciences, Fuzhou 350002, P. R. China

^2^ College of Chemistry, Fuzhou University, Fuzhou 350002, P. R. China

^3^ State Key Laboratory of Clean Energy Utilization, Zhejiang University, Hangzhou 310027, P. R. China

^4^ Geological Survey of Finland, P.O. Box 96, FI-02151, Espoo, Finland

^5^ Key Laboratory of Advanced Energy Materials Chemistry (Ministry of Education), Nankai University, Tianjin 300071, P. R. China

* Corresponding authors. E-mail: [huxiang@fjirsm.ac.cn](mailto:huxiang@fjirsm.ac.cn) (Xiang Hu); [junhua.xu@gtk.fi](mailto:junhua.xu@gtk.fi) (Junhua Xu); [wen@fjirsm.ac.cn](mailto:wen@fjirsm.ac.cn) (Zhenhai Wen)

**S1 Calculation of the b Value and Capacitive Contribution**

In cyclic voltammetry (CV) studies, the b-value is an important parameter used to describe the electrochemical kinetics process of electrode reactions. The magnitude of the b-value can help distinguish the dominant charge storage mechanism in the electrode reaction process, whether it is a capacitance-controlled process or a diffusion-controlled process. Generally speaking, the b-value ranges from 0.5 to 1. When b = 0.5, the reaction is mainly diffusion-controlled, conforming to the Randles-Sevcik equation for semi-infinite linear diffusion; when b=1, the reaction is mainly capacitance-controlled, such as the surface capacitance process. According to the formula [S1]:

$\text{i}\text{=a }\text{v}^{\text{b}}$ (S1)

Where i is the peak current, v is the scan rate, a is a constant related to the electrode material and the reaction system, and the b-value is obtained by performing logarithmic transformation on i and b

Taking the logarithm of both sides of the formula gives

$\log\text{(i)=b}\log\left( \text{v} \right)\text{+}\log\text{(a)}$ (S2)

By measuring the peak current i of the cyclic voltammetry curve at different scan rates v, and then performing linear fitting with log(v) as the abscissa and log(i) as the ordinate, the slope of the straight line is the b-value.

The ratios of Na^+^ capacitive contribution can be further quantitatively distinguished by separating the current response (i) at a specific potential (V) according to the following equations [S2]:

$\text{i}\left( \text{V} \right)\text{=}\text{k}_{\text{1}}\text{+}\text{k}_{\text{2}}\text{v}_{\text{2}}^{\text{1}}$ (S3)

$\frac{\text{i}\left( \text{V} \right)}{\text{v}_{\text{2}}^{\text{1}}}\text{=}\frac{\text{k}_{\text{1}}}{\text{v}_{\text{2}}^{\text{1}}}\text{+}\text{k}_{\text{2}}$ (S4)

where both k_1_ and k_2_ are constant values obtained from the slope and intercept of the i(V)/v^1/2^versus v^1/2^ plot, respectively. And k_1_v and k_2_v^1/2^ represent the capacitive contribution and ionic diffusion, respectively.

**S2 Galvanostatic intermittent titration (GITT) measurement**

According to the GITT theory, during the relaxation stage, the sodium ion diffusion coefficient (D_Na+_) can be calculated by the simplified Fick’s second law with the following formula [S3]:

$\text{D=}\frac{\text{4}}{\text{πτ}}{\text{(}\frac{\text{m}_{\text{B}}\text{V}_{\text{M}}}{\text{M}_{\text{B}}\text{S}}\text{)}}^{\text{2}}{\text{(}\frac{\text{∆}\text{E}_{\text{S}}}{\text{∆E}_{\text{τ}}}\text{)}}^{\text{2}}$ (S5)

Where $\text{τ}$ is the relaxation time,  $\text{V}_{\text{M}}$ is the molar volume of the sodium ion active material in the electrode material, $S$ is the contact area between the electrode and the electrolyte,  $\text{∆}\text{E}_{\text{S}}$ is the change in potential during the small current pulse stage, and  $\Delta E_{\tau}$ is the change in potential during the relaxation stage. $\text{m}_{\text{B}}$ is the the electrode active mass, $\text{M}_{\text{B}}$ (g mol^-1^) and $\text{V}_{\text{M}}$ (cm^3^ mol^-1^) are the molecular weight and molar volume of electrode material.

**S3 DFT parameters**

All the calculations are implemented by PWSCF codes contained in the Quantum ESPRESSO distribution [S4]. Spin-polarized DFT calculations were performed with periodic super-cells under the generalized gradient approximation (GGA) using the Perdew-Burke-Ernzerhof (PBE) functional for exchange-correlation and the ultrasoft pseudopotentials for nuclei and core electrons. The Kohn-Sham orbitals were expanded in a plane-wave basis set with a kinetic energy cutoff of 30 Ry and the charge-density cutoff of 300 Ry. The Fermi-surface effects has been treated by the smearing technique of Methfessel and Paxton, using a smearing parameter of 0.02 Ry. To further enhance accuracy, the dispersion correction was consistently incorporated across all calculations, employing the semiempirical zero damping D3 method advanced by Grimme [S5].

The Brillouin-zones were sampled with a k-point mesh of 1×1×1. The calculation model of MnS_0.5_Se_0.5_ and MnS was constructed with 3×3 lateral periodicity. MnSe in the calculation model were constructed with 1×1 lateral periodicity. (100) surface was chosen for MnS_0.5_Se_0.5_, MnS and MnSe to represent the exposed surfaces.

The diffusion barrier for Na atom was determined by nudged elastic band (NEB). The vacuum layer was ∼ 15 Å to remove the interaction of adjacent atomic slabs in the z direction.

**S4 Details for adsorption energies calculation and charge density difference**

The adsorption energy ΔEa can be calculated by the following equation:

$\text{∆}\text{E}_{\text{a}}\text{=}\text{E}_{\text{tot}}\text{-}\text{E}_{\text{N}_{\text{a}}}\text{-}\text{E}_{\text{str}}$ (S6)

where $\text{E}_{\text{tot}}$ is the total energy of compound obtained from DFT calculations, $\text{E}_{\text{tot}}$ is the energy of Na atoms and $\text{E}_{\text{str}}$ is the energy of each structure. Electron density difference was calculated by subtracting the charge densities of Na atom and each configuration from the corresponding compounds.

The charge density difference can be used to analyze the bonding process or the charge transfer before and after structural relaxation. The charge density difference of system can be calculated by the following equation:

$\text{∆ρ=}\text{ρ}_{\text{AB}}\text{-}\text{ρ}_{\text{A}}\text{-}\text{ρ}_{\text{B}}$ (S7)

Where $\text{ρ}_{\text{AB}}$ is the composition, $\text{ρ}_{\text{A}}$ is base and $\text{ρ}_{\text{B}}$ is absorabte. In calculation of thelatter two quantities, the atomic positions are fixed as those they have in the AB system.

**S5 Supplementary Figures and Tables**


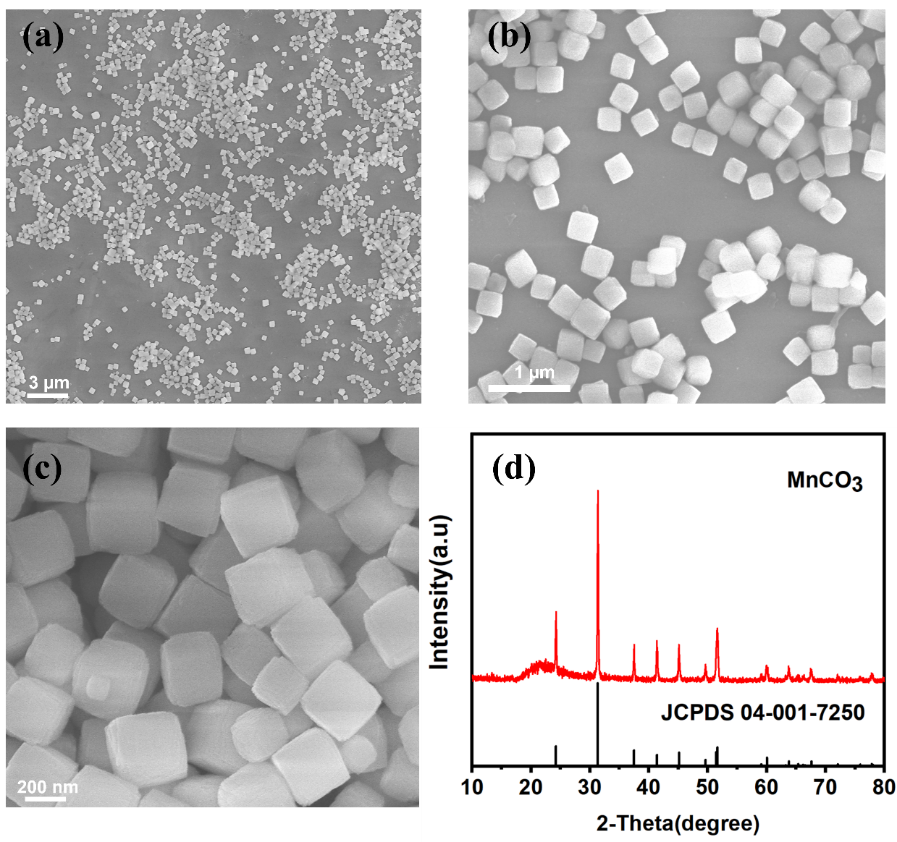


**Fig. S1 (a-c)** SEM images, **(d)** XRD pattern of MnCO_3_ nanocubes


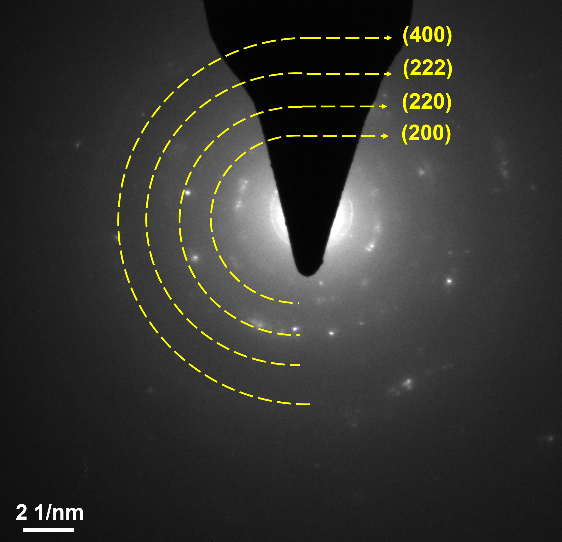


**Fig. S2** The selected area electron diﬀraction (SAED) pattern of MnS0.5Se0.5@N-CNF


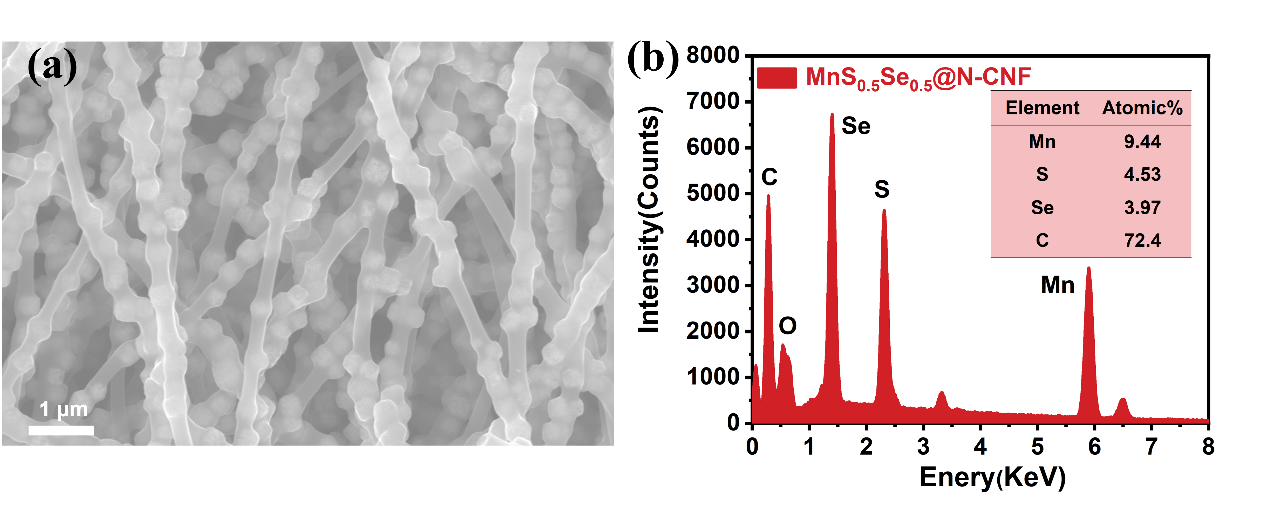


**Fig. S3 (a)** SEM image, **(b)** the corresponding EDX spectrum and table (inset) illustrating the element content conta of MnS_0.5_Se_0.5_@N-CNF


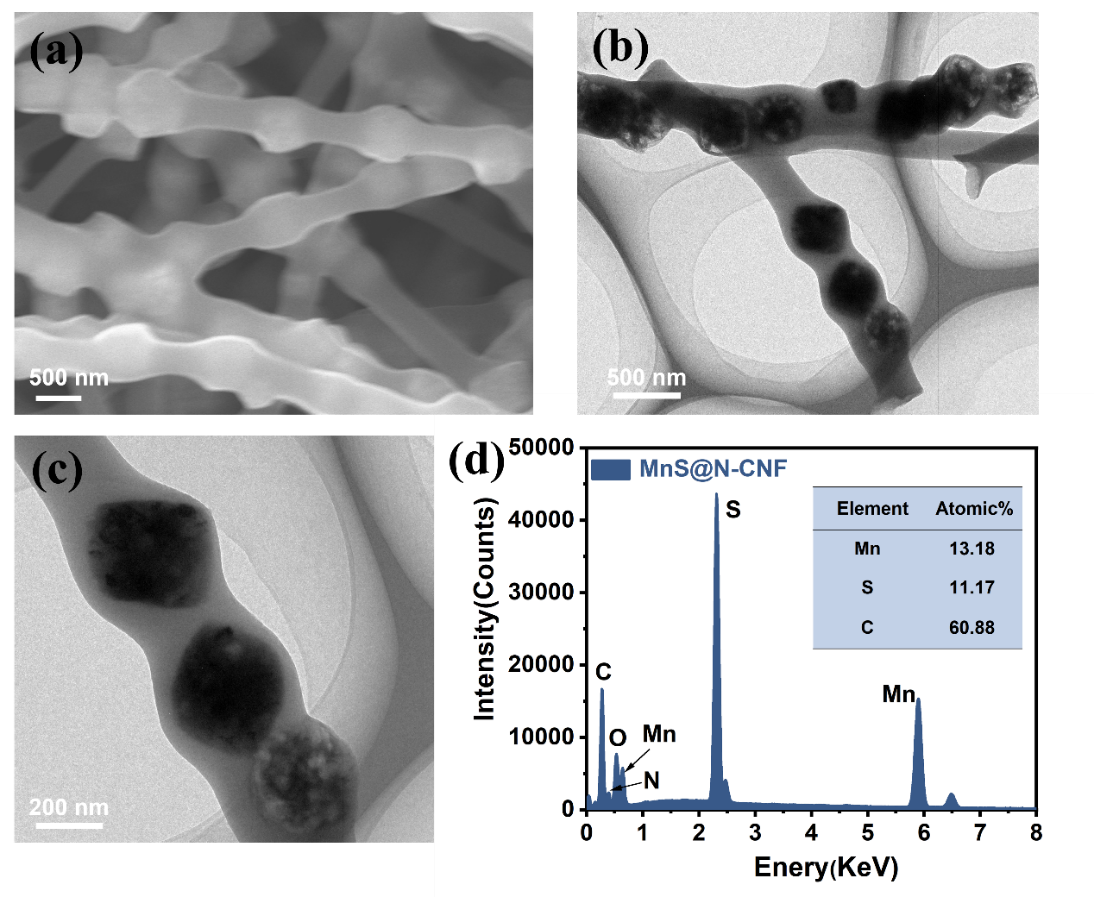


**Fig. S4 (a)** SEM image, **(b, c)** TEM images, **(d)** the corresponding EDX spectrum and

table (inset) illustrating the element content conta of MnS@N-CNF


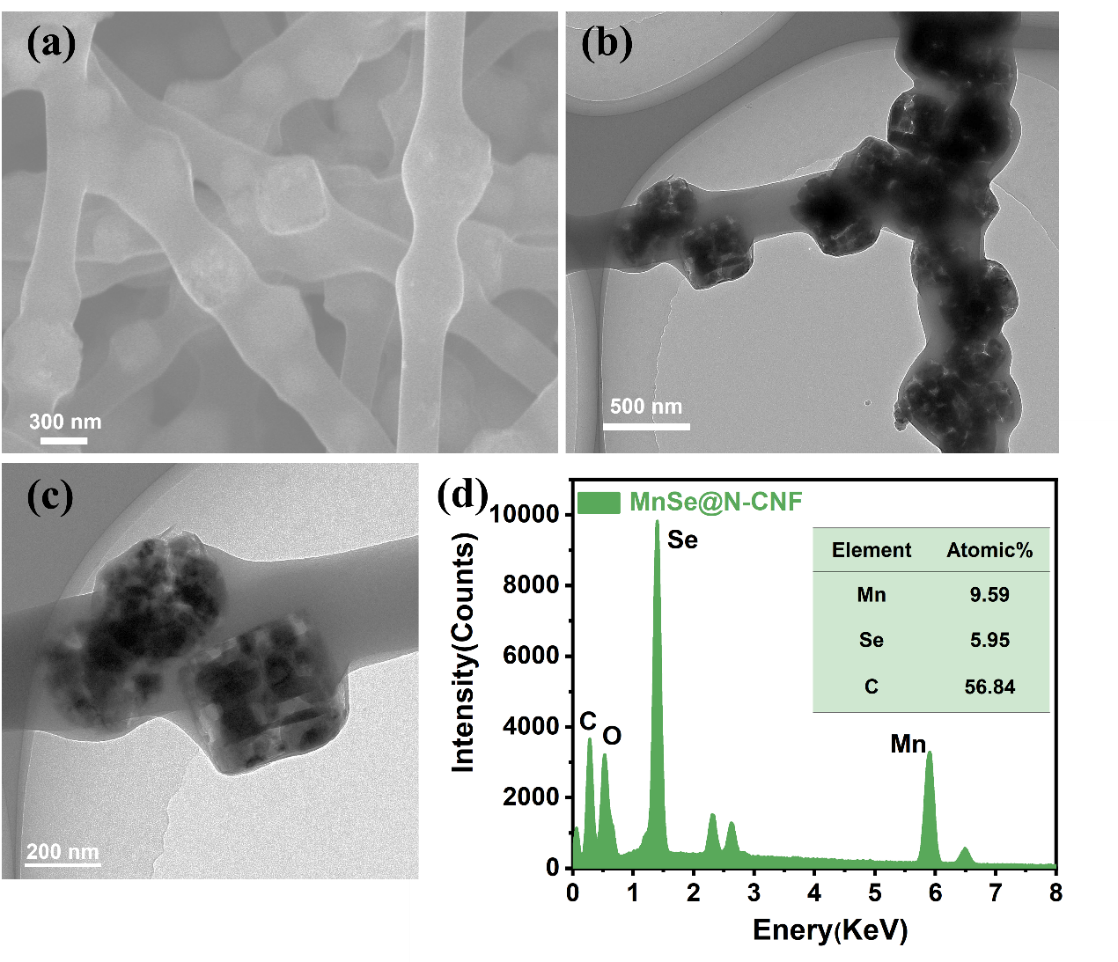


**Fig. S5** **(a)** SEM image, **(b, c)** TEM images, **(d)** the corresponding EDX spectrum and

table (inset) illustrating the element content conta of MnSe@N-CNF


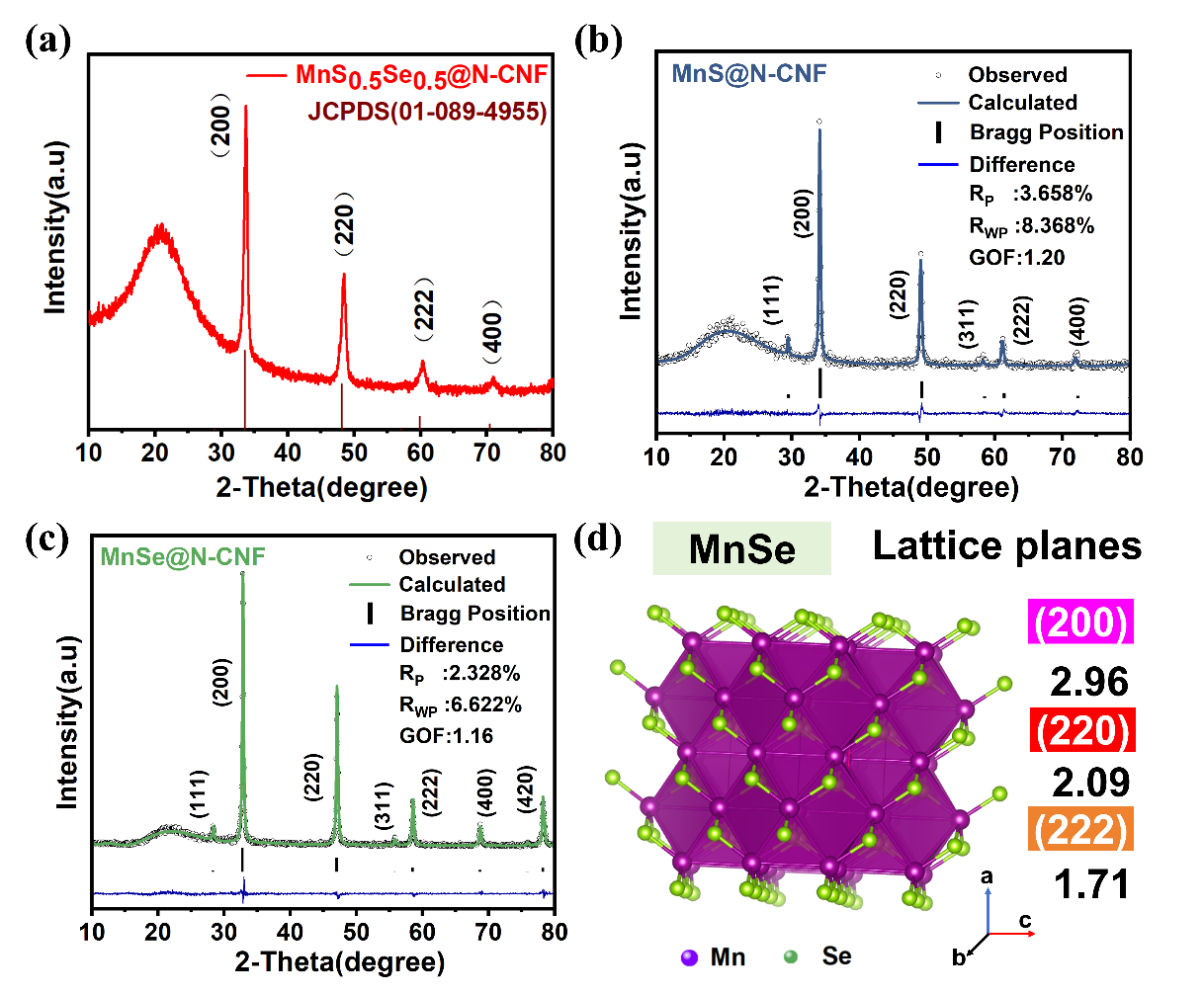


**Fig. S6 (a)** XRD patterns of the MnS_0.5_Se_0.5_@N-CNF, **(b, c)** Rietveld-refined XRD result of the MnS@N-CNF and MnSe@N-CNF. **(d)** Crystal structures of MnSe@N-CNF


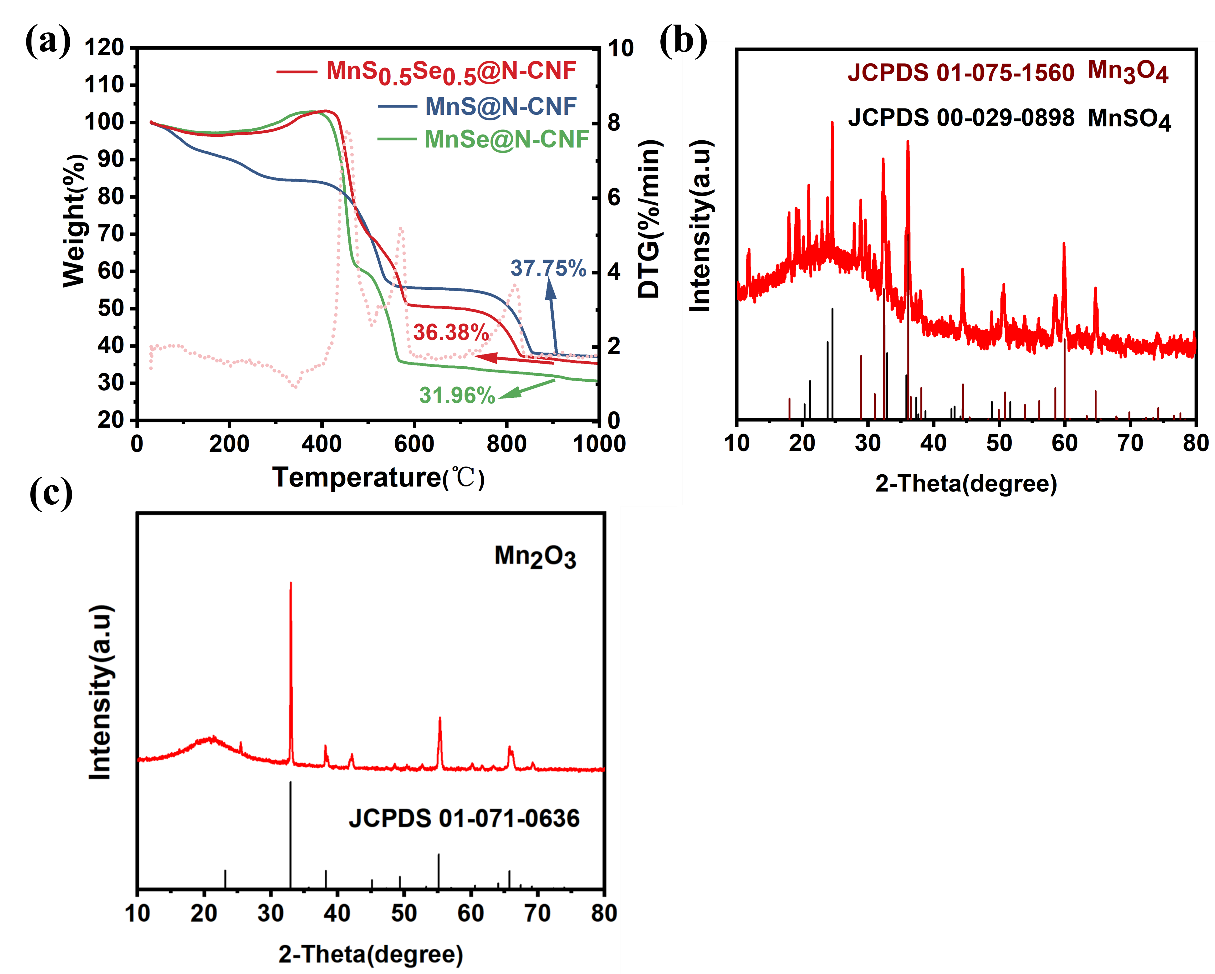


**Fig. S7 (a)** TGA curves, **(b, c)** XRD pattern of the residual material after TGA test at 600 ℃ and 900 ℃ of MnS_0.5_Se_0.5_@N-CNF composite

**TGA** **analysis:**

The weight percentage of carbon nanofiber (N-CNF) in the MnS_0.5_Se_0.5_@N-CNF composites was analyzed using thermogravimetric analysis (TGA). As depicted in Fig. S7, a minor weight loss below 300 °C was attributed to the evaporation of absorbed water on the surface of the samples. The weight gain observed between 300 and 500 °C was due to the formation of manganese sulfate (MnSO_4_) and manganese oxide (Mn_3_O_4_). The following rapid mass loss occurring between 450 to 600 °C signified the complete combustion of N-CNF in the composite, while the second weight reduction between 700 and 900 °C corresponded to the transformation of MnSO_4_ and Mn_3_O_4_ into Mn_2_O_3_. The exothermic peak in the differential thermogravimetry curve (red line in Fig. S7a) aligns with the weight loss curve, indicating chemical reactions and phase transitions. Note that SeO_2_, SO_2_ and CO_2_ would be evaporated completely after the TGA measurement. The total reaction can be simply written as:

$$\text{C+}\text{O}_{\text{2}}\text{→C}\text{O}_{\text{2}}$$

$$\text{4Mn}\text{S}_{\text{0.5}}\text{Se}_{\text{0.5}}\text{+9}\text{O}_{\text{2}}\text{→}\text{MnS}\text{O}_{\text{4}}\text{+}\text{Mn}_{\text{3}}\text{O}_{\text{4}}\text{+3S}\text{O}_{\text{2}}\text{+2Se}\text{O}_{\text{2}}$$

$$\text{MnS}\text{O}_{\text{4}}\text{+}\text{Mn}_{\text{3}}\text{O}_{\text{4}}\text{→}\text{2Mn}_{\text{2}}\text{O}_{\text{3}}\text{+S}\text{O}_{\text{2}}$$

Therefore, the MnS_0.5_Se_0.5_@N-CNF composite can be calculated according to the following equation:

$$\text{M}\text{n}\text{S}\text{0.5}\text{Se}\text{0.5}\left( \text{wt\%} \right)\text{=}\frac{\text{molecular weight of Mn}\text{S}\text{0.5}\text{Se}\text{0.5}}{\text{molecular weight of }\text{Mn}_{\text{2}}\text{O}_{\text{3}}}\text{×}\frac{\text{final weight of }\text{Mn}_{\text{2}}\text{O}_{\text{3}}}{\text{initial weight of Mn}\text{S}\text{0.5}\text{Se}\text{0.5}}\text{×100\%}$$

Based on the final residual weight, which was verified by the XRD pattern (Fig. S7b-c), the mass content of MnS_0.5_Se_0.5_ in MnS_0.5_Se_0.5_@N-CNF composite is calculated as about 88.9 %, and the corresponding weight percentage of N-CNF in the sample is estimated to be 11.1 %. Given the theoretical capacity of carbon at 372 mAh g^-1^, the capacity contribution from carbon matrix (N-CNF) in the MnS_0.5_Se_0.5_@N-CNF composite is only 41.3 mAh g^-1^, which can be negligible.

From the TGA curves (Fig. S7a), the weight loss observed before 200 °C can be attributed to the evaporation of moisture adsorbed on the surface of the MnS@N-CNF and MnSe@N-CNF samples. Subsequently, a stepwise weight loss occurs in the range from 400 to 900 °C, indicative of carbon burnout and the oxidation of MnS and MnSe into Mn_2_O_3_, SeO_2_, SO_2_ and CO_2_, respectively. It is worth noting that SeO_2_, SO_2_ and CO_2_ would evaporate completely following the TGA measurement. The total reaction can be simply written as:

$$\text{C+}\text{O}_{\text{2}}\text{→C}\text{O}_{\text{2}}$$

$$\text{2MnS+}\frac{\text{7}}{\text{2}}\text{O}_{\text{2}}\text{→}\text{Mn}_{\text{2}}\text{O}_{\text{3}}\text{+2S}\text{O}_{\text{2}}$$

$$\text{2MnSe+}\frac{\text{7}}{\text{2}}\text{O}_{\text{2}}\text{→}\text{Mn}_{\text{2}}\text{O}_{\text{3}}\text{+2Se}\text{O}_{\text{2}}$$

Therefore, the MnS@N-CNF and MnSe@N-CNF composite can be calculated according to the following equation:

$$\text{M}\text{n}\text{S}\text{ }\left( \text{wt}\text{\%} \right)\text{=}\frac{\text{molecular weight of Mn}\text{S}}{\text{molecular weight of }\text{Mn}_{\text{2}}\text{O}_{\text{3}}}\text{×}\frac{\text{final weight of }\text{Mn}_{\text{2}}\text{O}_{\text{3}}}{\text{initial weight of MnS}}\text{×100\%}$$

$$\text{M}\text{n}\text{S}\text{e}\text{ }\left( \text{wt}\text{\%} \right)\text{=}\frac{\text{molecular weight of Mn}\text{Se}}{\text{molecular weight of }\text{Mn}_{\text{2}}\text{O}_{\text{3}}}\text{×}\frac{\text{final weight of }\text{Mn}_{\text{2}}\text{O}_{\text{3}}}{\text{initial weight of MnS}\text{e}}\text{×100\%}$$

Based on the TGA result, the mass content of MnS in MnS@N-CNF composite is calculated as about 68.5%, and the corresponding weight percentage of N-CNF in the sample is estimated to be 31.5%. The mass content of MnSe in MnSe@N-CNF composite is calculated as about 57.7%, and the corresponding weight percentage of N-CNF in the sample is estimated to be 42.3%.


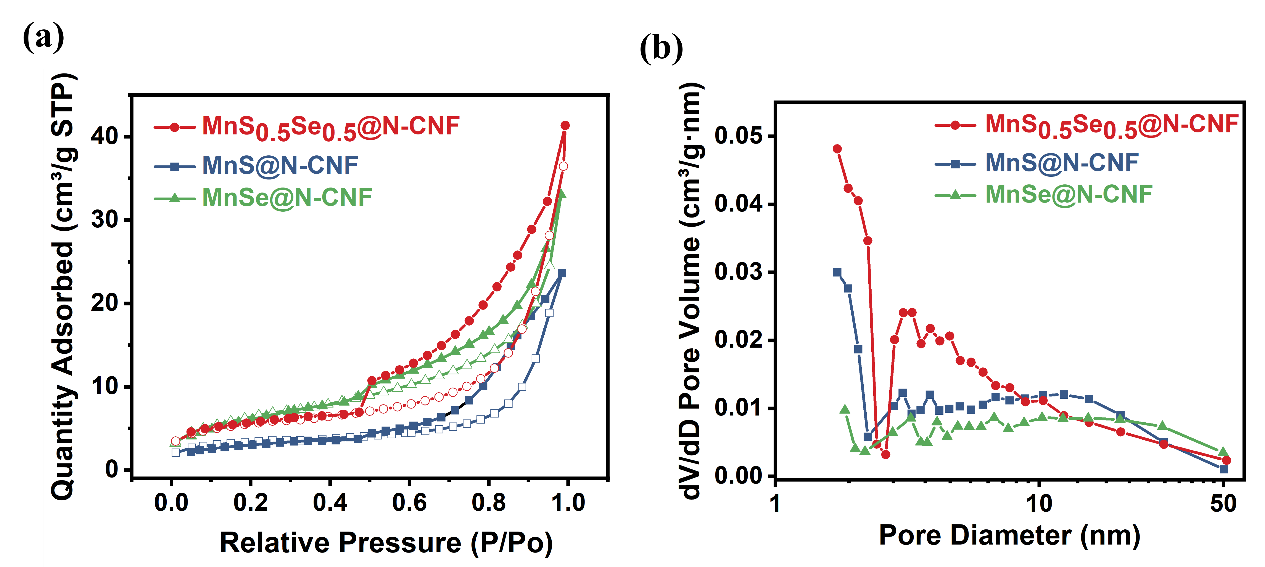


**Fig. S8 (a)** Nitrogen adsorption-desorption isothermal and **(b)** pore size distributions curves of MnS_0.5_Se_0.5_@N-CNF, MnS@N-CNF and MnSe@N-CNF


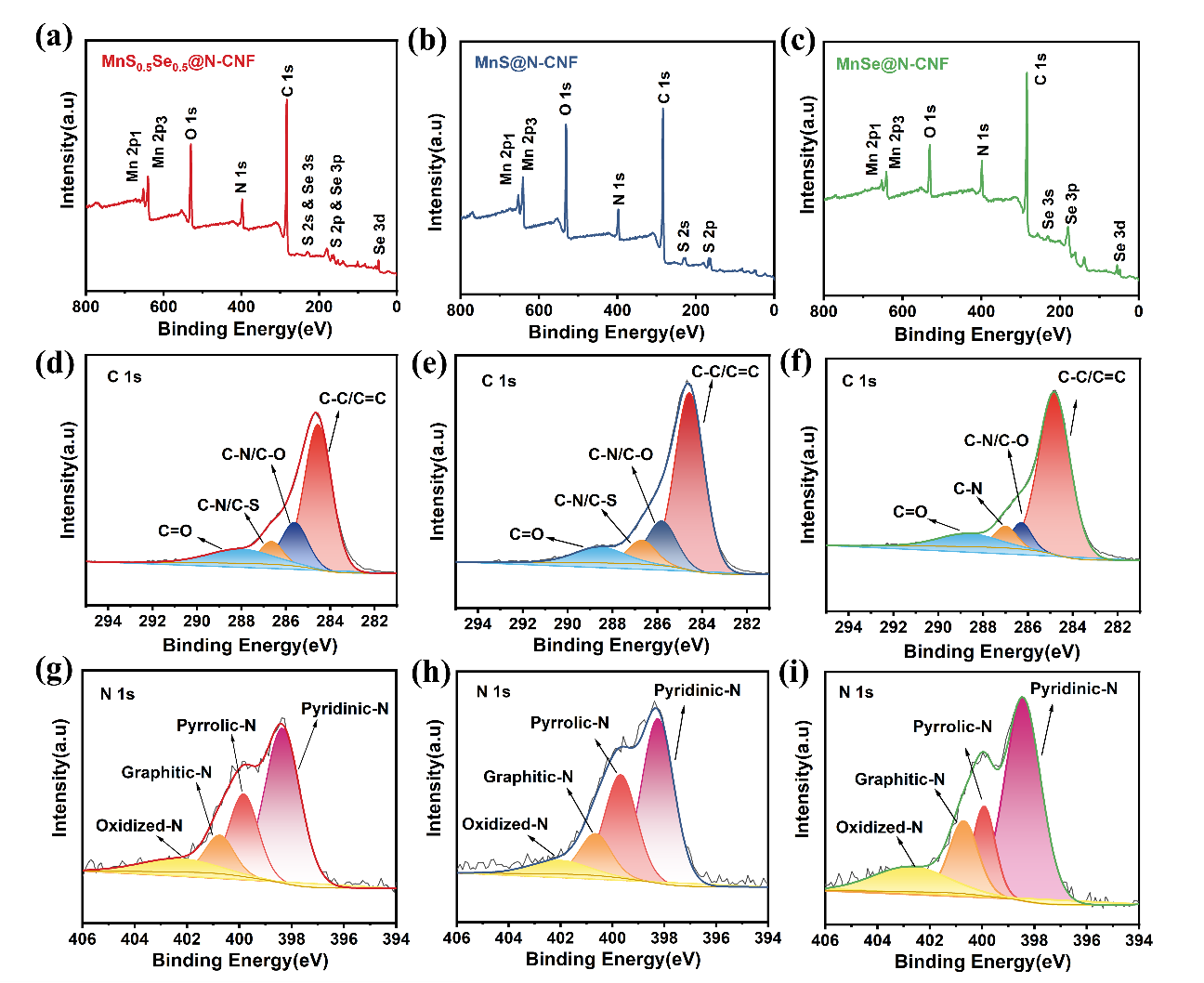


**Fig. S9** The survey XPS spectrum of **(a)** MnS_0.5_Se_0.5_@N-CNF, **(b)** MnS@N-CNF and **(c)** MnSe@N-CNF. The high-resolution C 1s spectra of **(d)** MnS_0.5_Se_0.5_@N-CNF, **(e)** MnS@N-CNF and **(f)** MnSe@N-CNF. The high-resolution N 1s spectra of **(g)** MnS_0.5_Se_0.5_@N-CNF, **(h)** MnS@N-CNF and **(i)** MnSe@N-CNF


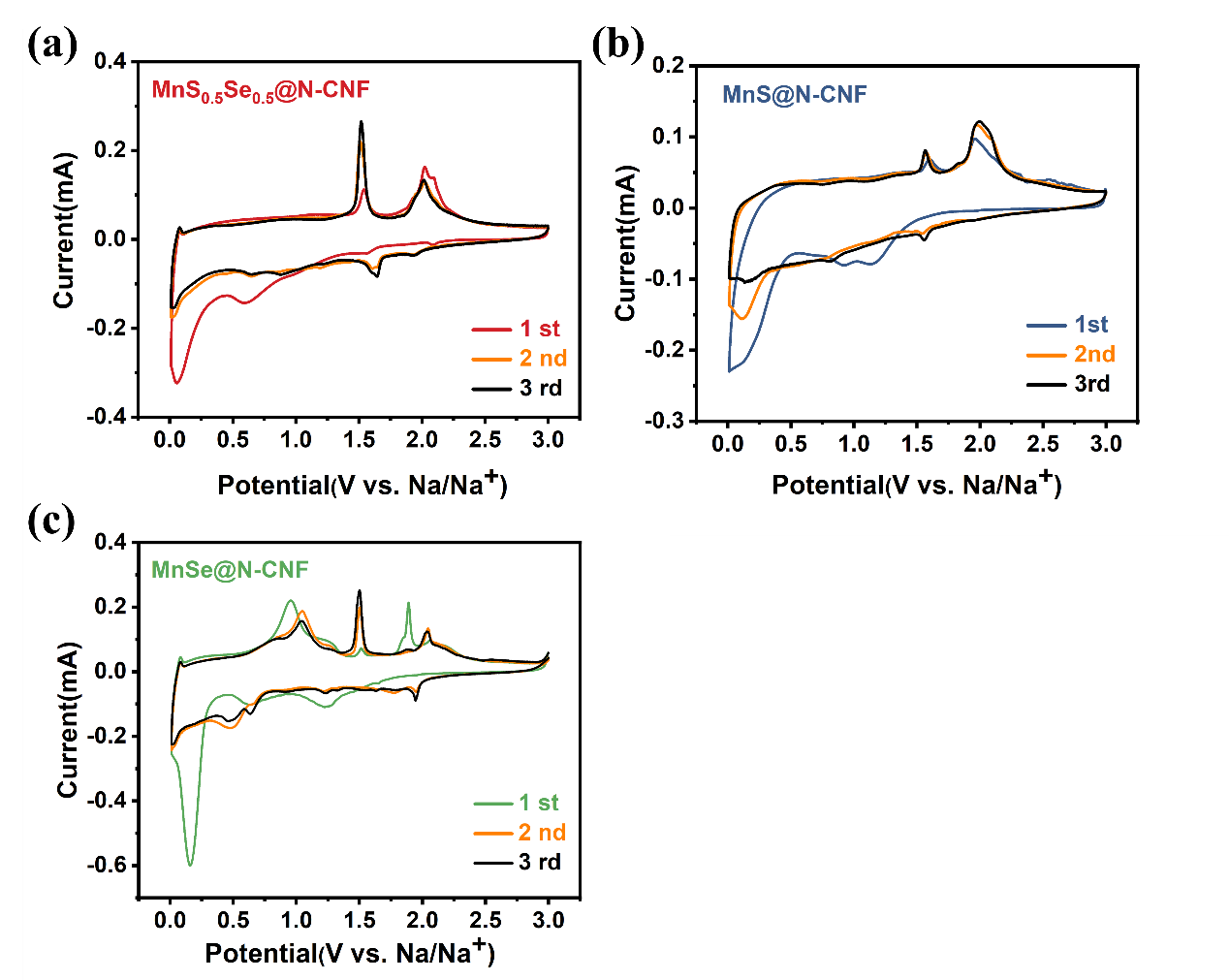


**Fig. S10** CV curves of the **(a)** MnS_0.5_Se_0.5_@N-CNF, **(b)** MnS@N-CNF and **(c)** MnSe@N-CNF electrodes for the first cycles at a scan rate of 0.1 mV s^-1^


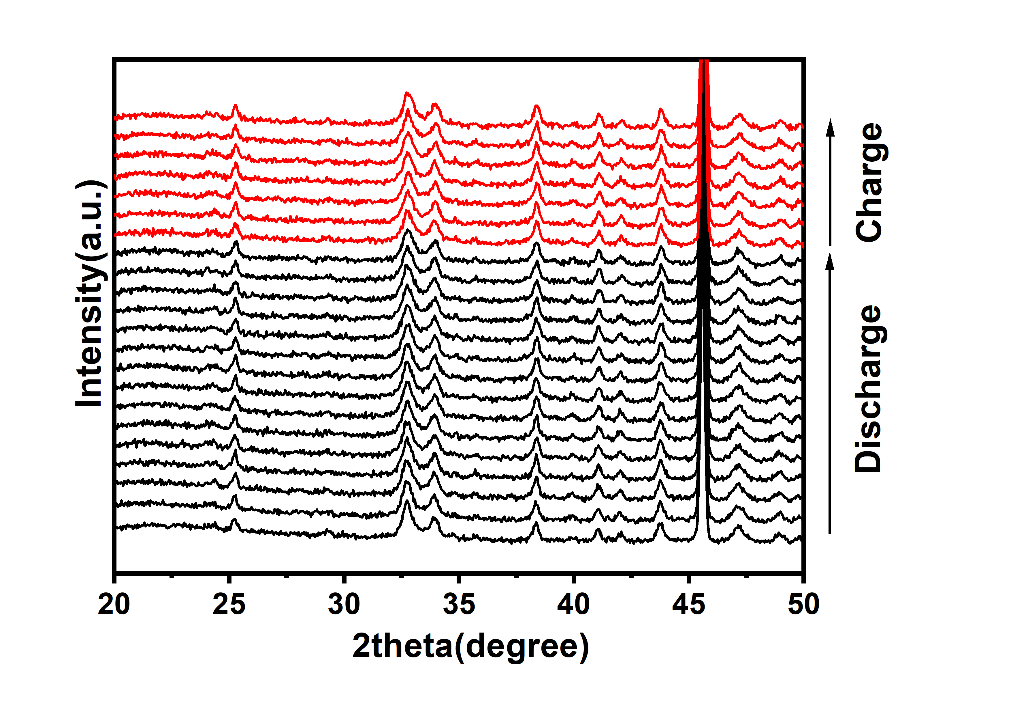


**Fig. S11** In-situ XRD patterns of MnS_0.5_Se_0.5_@N-CNF electrode during the initial cycle


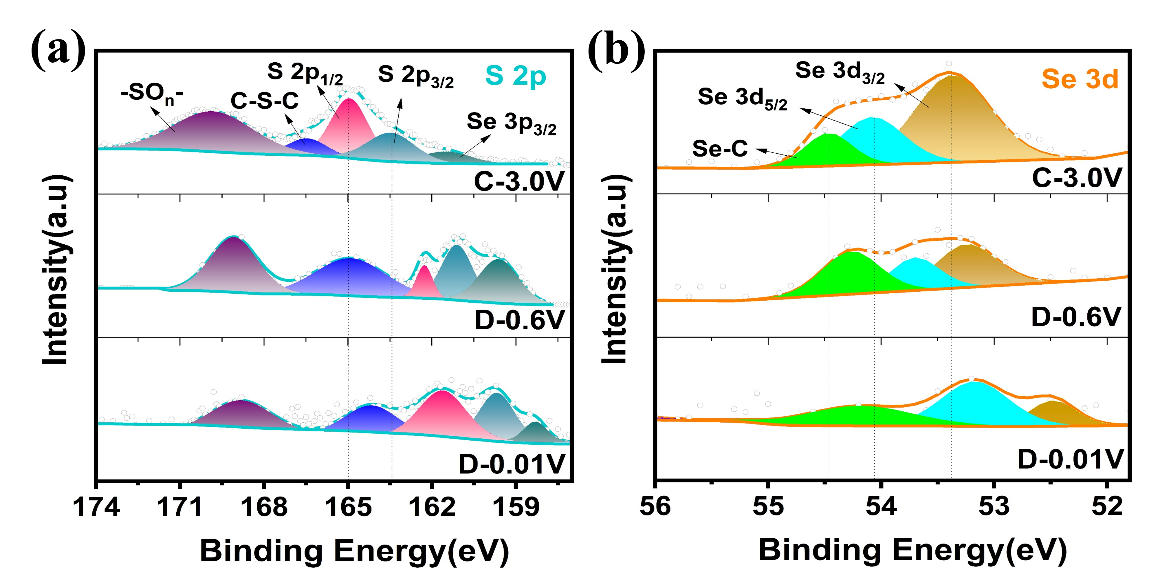


**Fig. S12** The high-resolution spectrum of **(a)** S 2p and **(b)** Se 3d at different potentials for the MnS_0.5_Se_0.5_@N-CNF electrode during the initial cycle


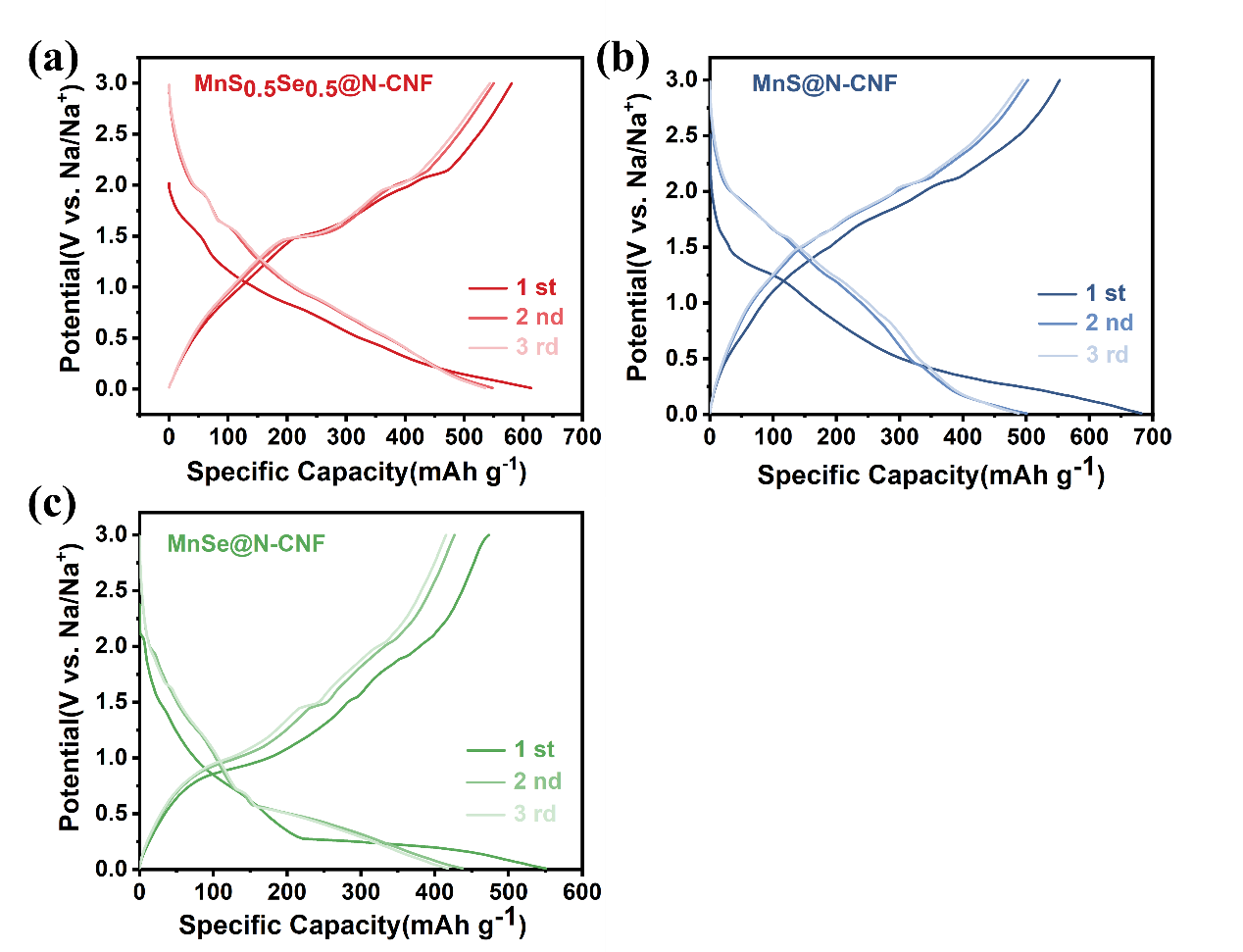


**Fig. S13** Galvanostatic charge/discharge voltage profiles of the **(a)** MnS_0.5_Se_0.5_@N-CNF, **(b)** MnS@N-CNF and **(c)** MnSe@N-CNF electrodes for the first three cycles at a current density of 0.1A g^-1^


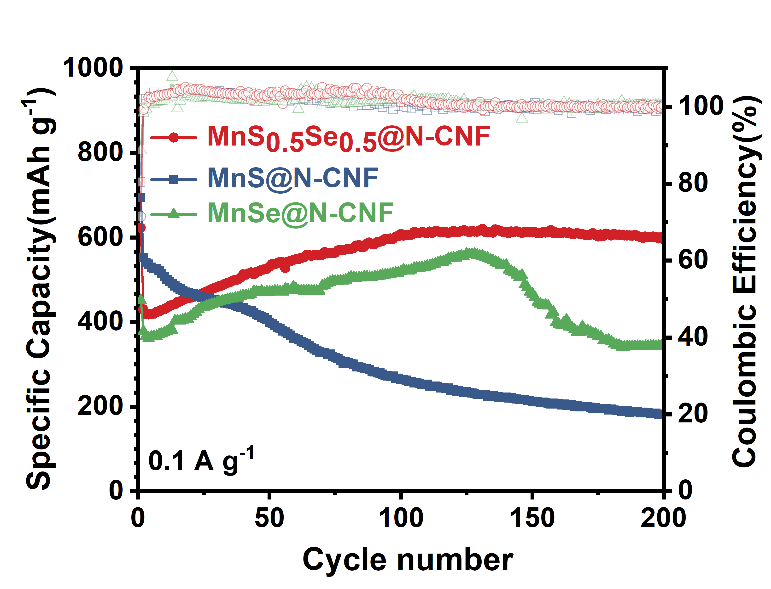


**Fig. S14** Cycling stability of MnS_0.5_Se_0.5_@N-CNF, MnS@N-CNF and MnSe@N-CNF at 0.1 A g^-1^


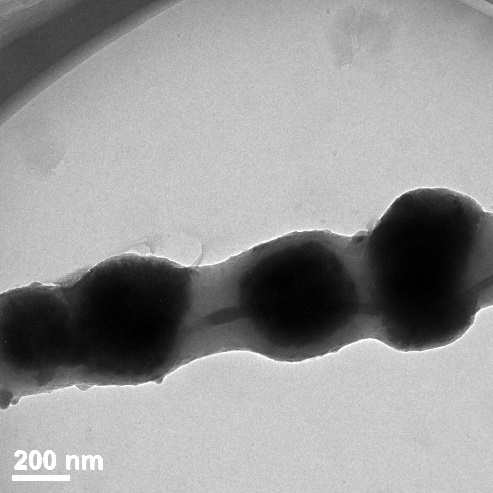


**Fig. S15** The TEM image of the electrode sheets of the MnS_0.5_Se_0.5_@N-CNF sample after 200 cycles of charge and discharge at 0.1 A g^-1^


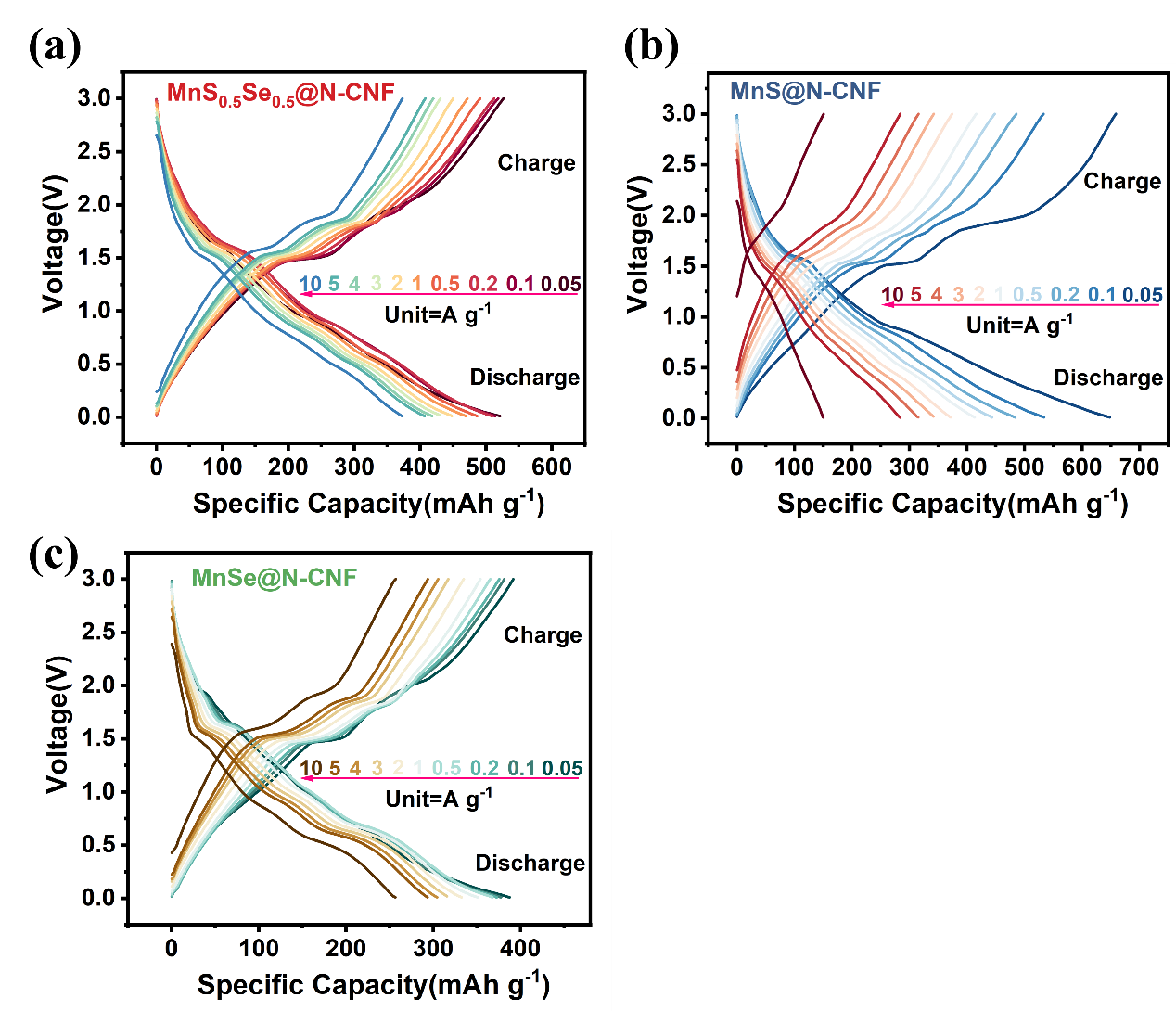


**Fig. S16** Charge-discharge proﬁles of the **(a)** MnS_0.5_Se_0.5_@N-CNF, **(b)** MnS@N-CNF and **(c)** MnSe@N-CNF electrodes for SIBs at various current densities

*
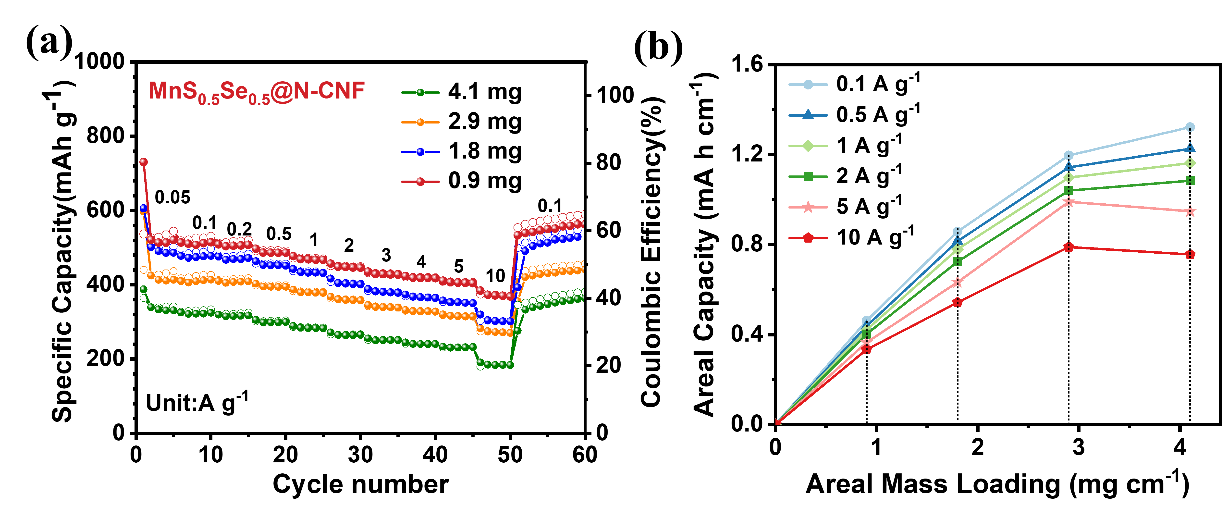
*

**Fig. S17 (a)** Comparison of the rate performance between 0.05 A g^-1^ and 10 A g^-1^ for MnS_0.5_Se_0.5_@N-CNF electrodes under different mass loadings (0.9, 1.8, 2.9 and 4.1 mg cm^-2^). **(b)** The correlation of areal capacity with mass loading at various rates for the MnS_0.5_Se_0.5_@N-CNF electrode


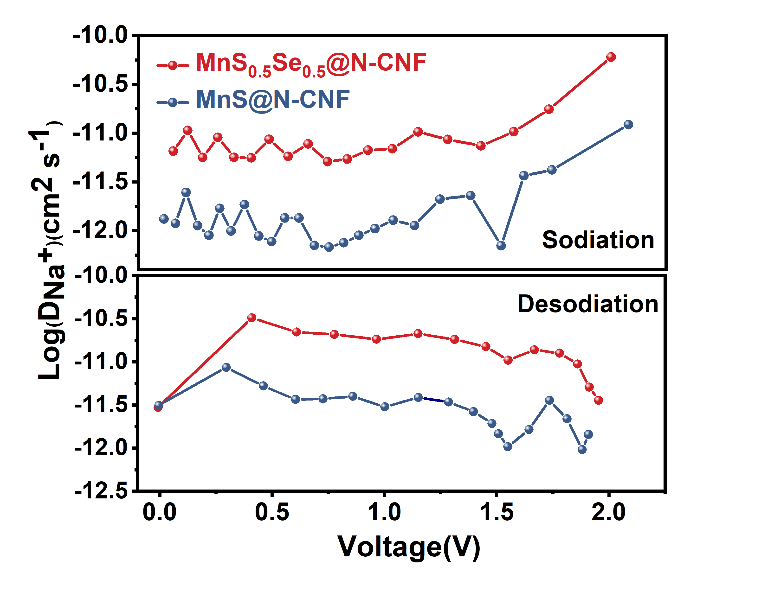


**Fig. S18** Na^+^ diffusion coefficients of the MnS_0.5_Se_0.5_@N-CNF and MnS@N-CNF and at 0.05 A g^-1^ after the 3rd cycles during the sodiation and desodiation process


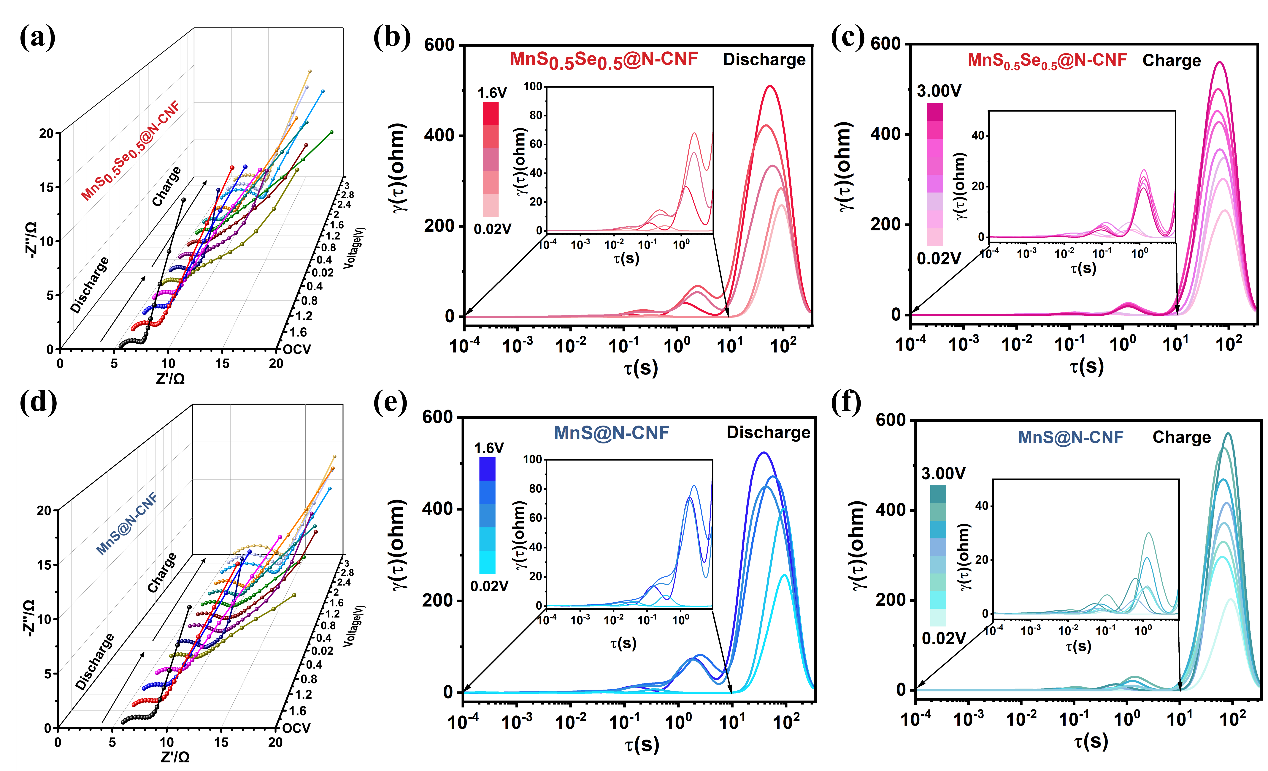


**Fig. S19** **(a)** In situ EIS proﬁles of the MnS_0.5_Se_0.5_@N-CNF and (d) MnS@N-CNF electrodes recorded during the initial cycle at a current rate of 50 mA g^−1^. **(b, c)** DRT calculated from EIS measurements of MnS_0.5_Se_0.5_@N-CNF at different potentials. **(e, f)** DRT calculated from EIS measurements of MnS@N-CNF at different potentials


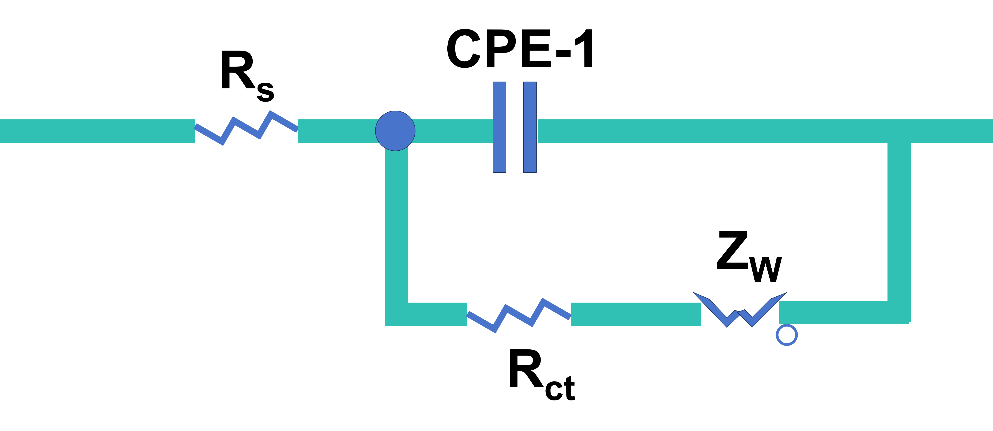


**Fig. S20** Equivalent circuits of Electrochemical impedance spectra (EIS)


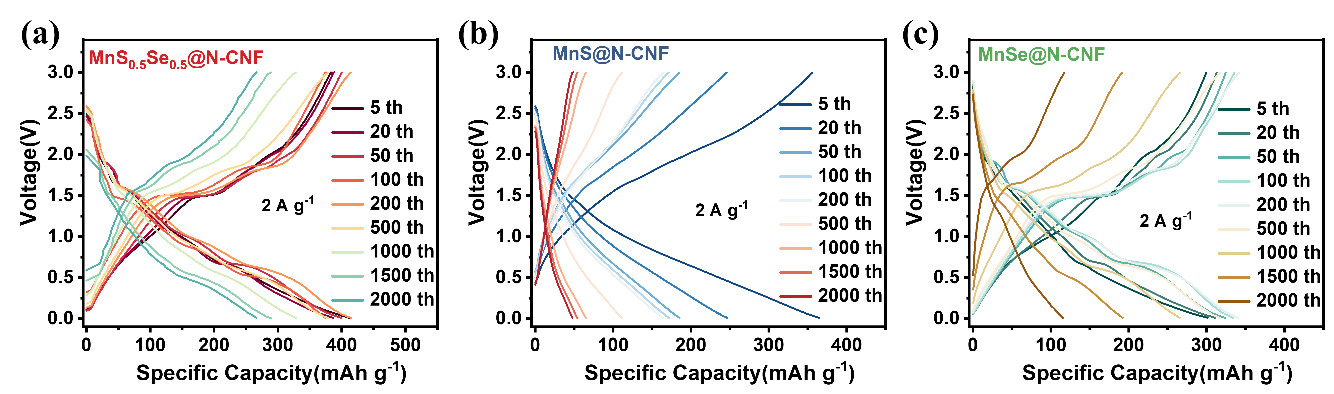


**Fig. S21** The charge-discharge profile of different cycles including the 2000th cycle for **(a)** MnS_0.5_Se_0.5_@N-CNF, **(b)** MnS@N-CNF and **(c)** MnSe@N-CNF electrodes


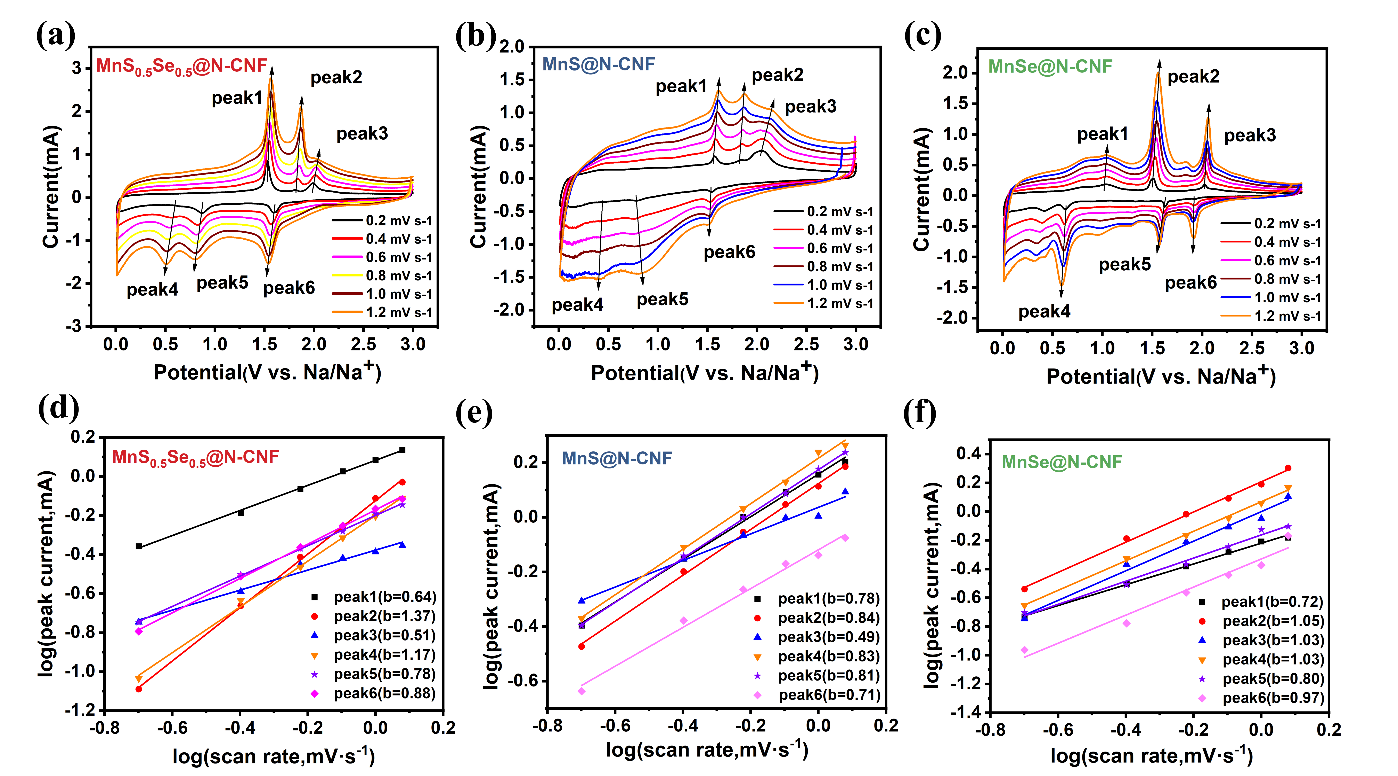


**Fig. S22** CV curves at various scan rates from 0.2 to 1.2 mV s^-1^ and linear relationships between logarithm currents and logarithm sweep rate for **(a, d)** MnS_0.5_Se_0.5_@N-CNF, **(b, e)** MnS@N-CNF and **(c, f)** MnSe@N-CNF electrodes


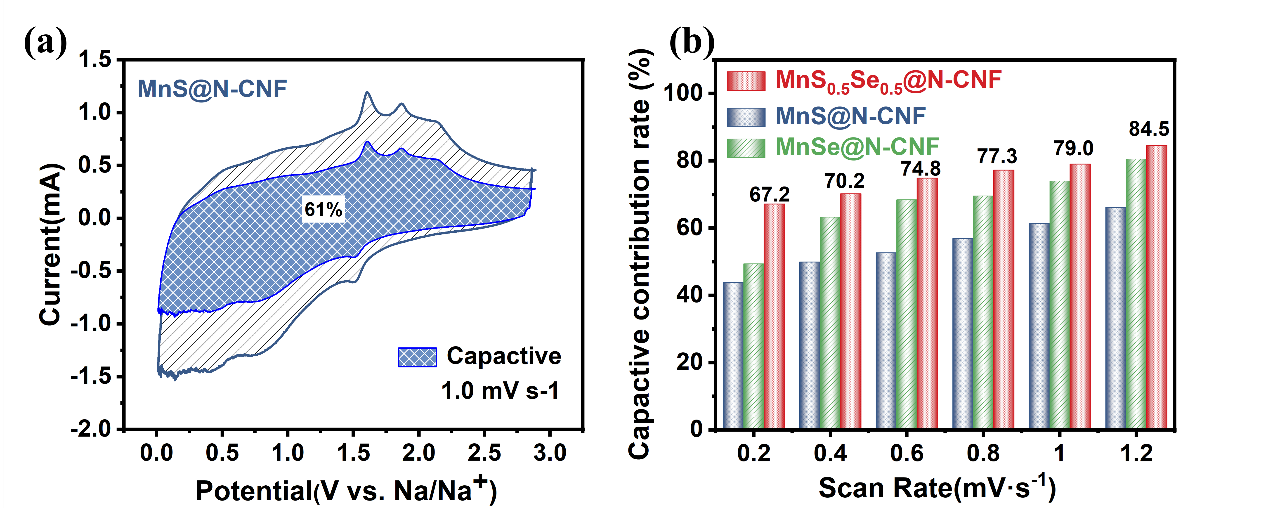


**Fig. S23** **(a)** Capacitive contribution at 1.0 mV s^-1^ of the MnS@N-CNF electrode. **(b)** Calculated capacitive contributions at different sweep rate for the MnS_0.5_Se_0.5_@N-CNF, MnS@N-CNF and MnSe@N-CNF electrodes


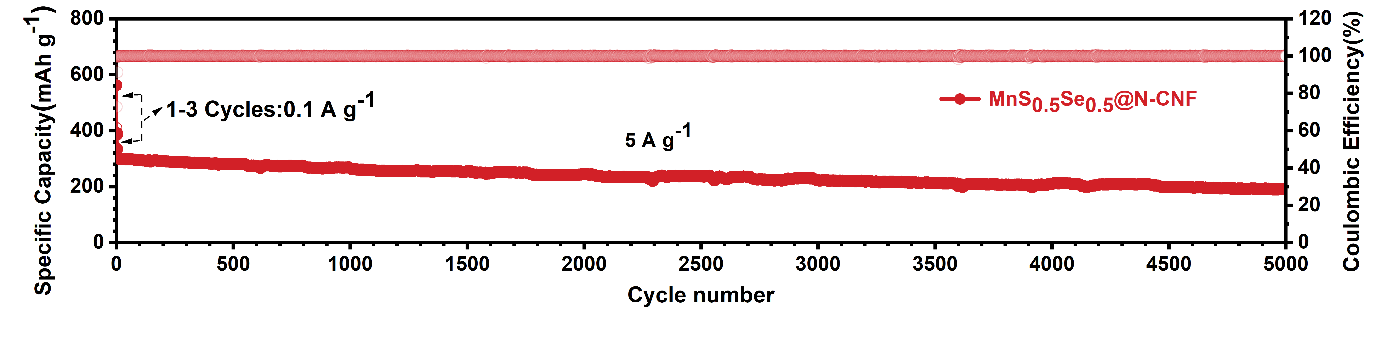


**Fig. S24** Long cycling performance at 5 A g^-1^ of the MnS_0.5_Se_0.5_@N-CNF electrodes


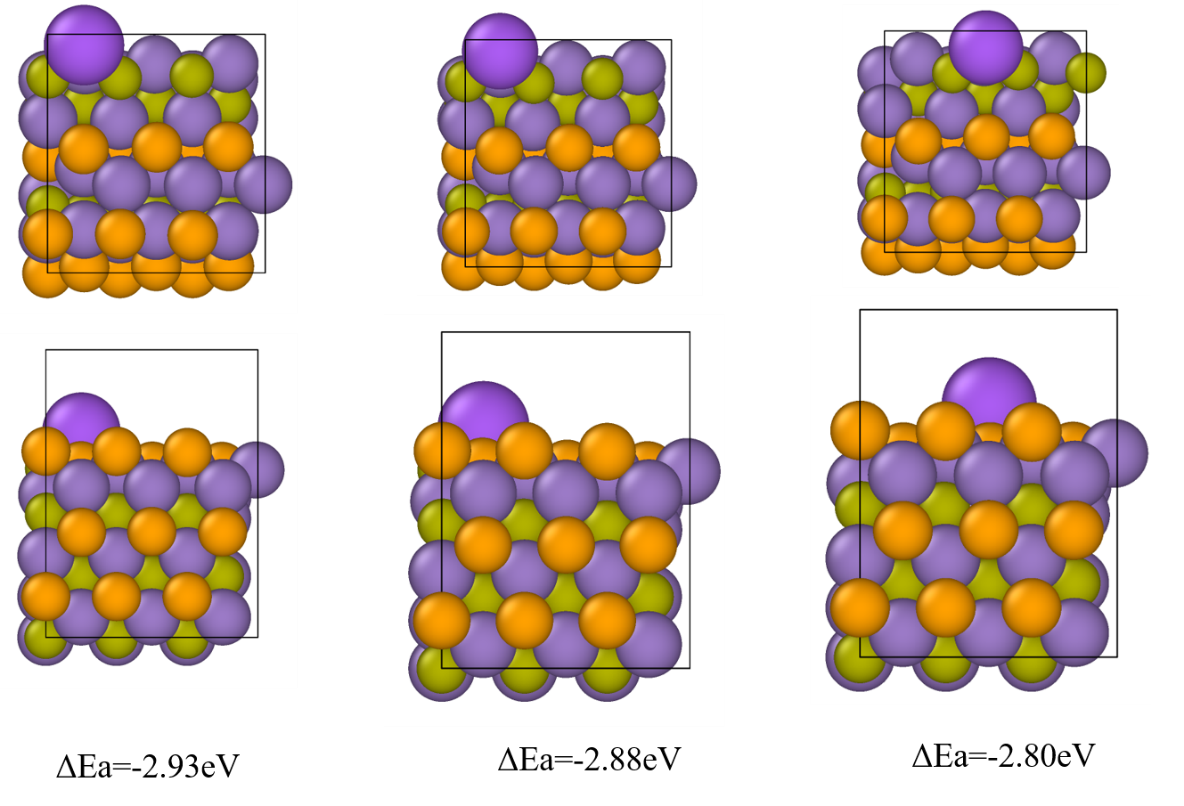


**Fig. S25** Top and side view of the simulations for one Na ion adsorbed on the MnS_0.5_Se_0.5_ as well as corresponding ΔEa


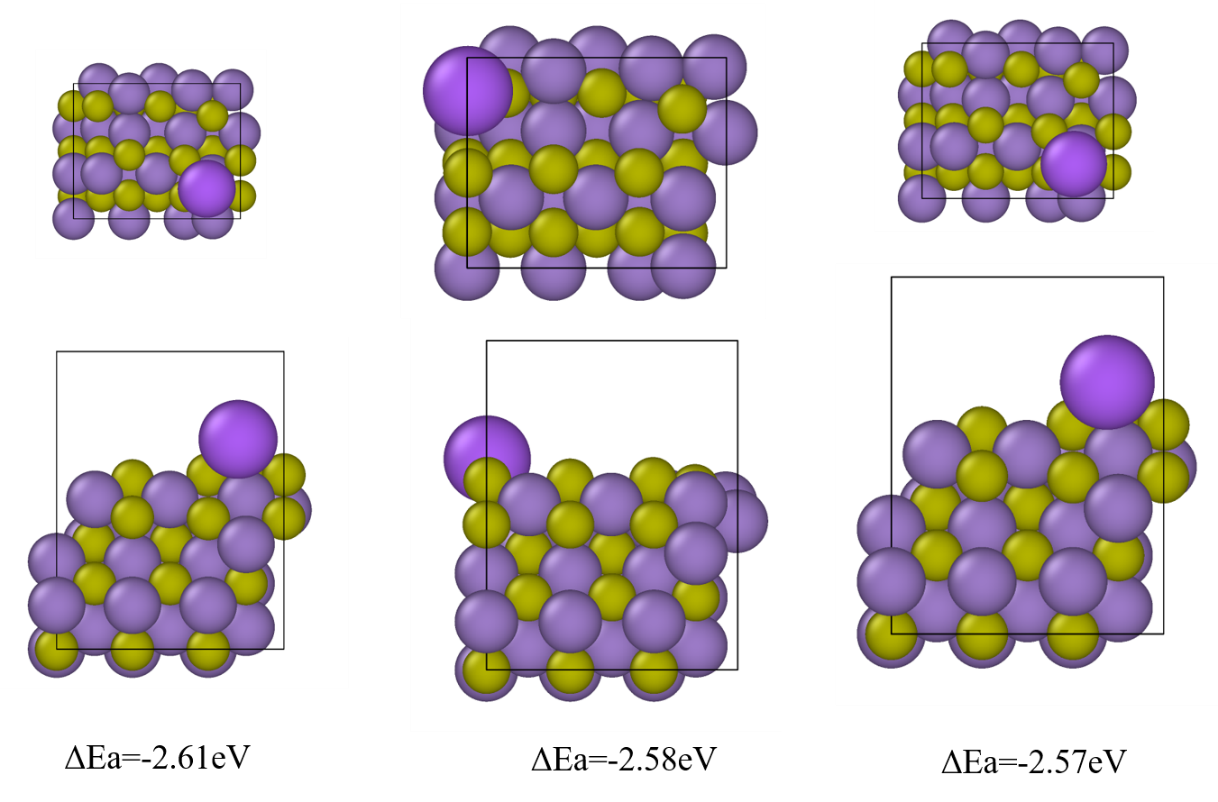


**Fig. S26** Top and side view of the simulations for one Na ion adsorbed on the MnS as well as corresponding ΔEa


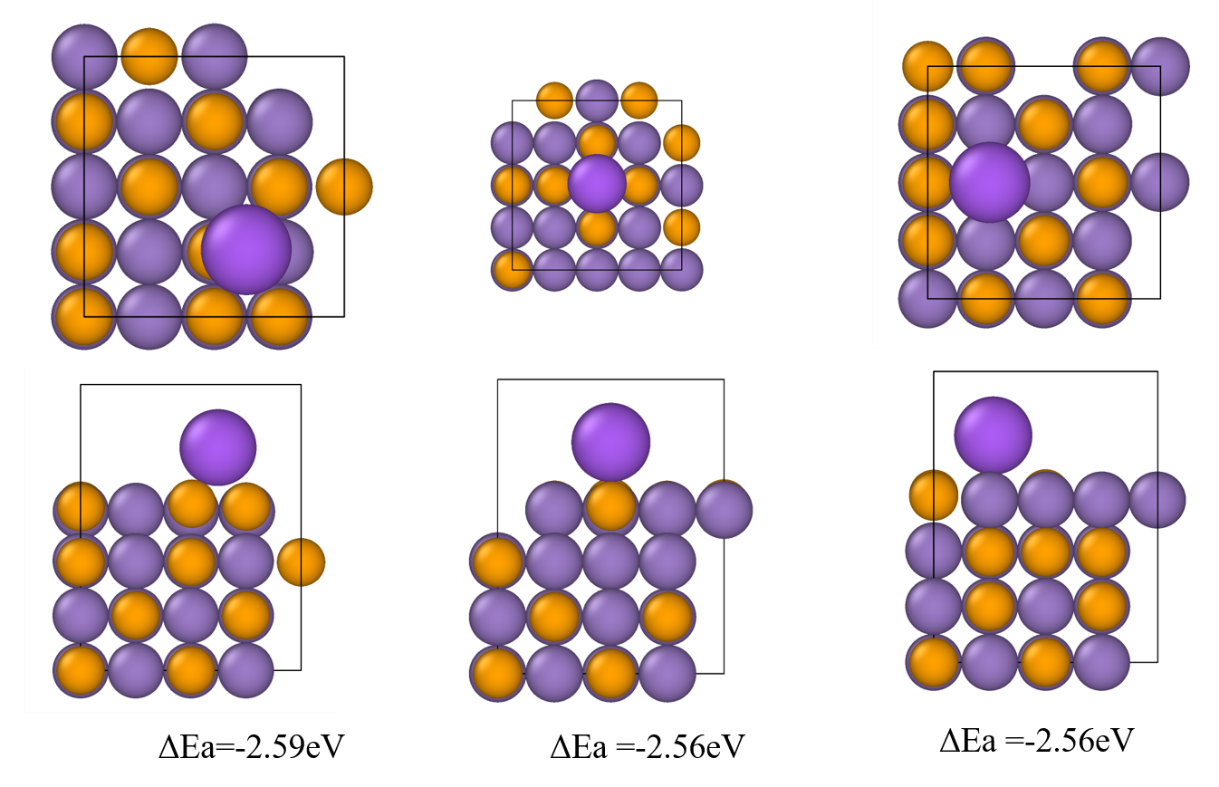


**Fig. S27** Top and side view of the simulations for one Na ion adsorbed on the MnSe as well as corresponding ΔEa


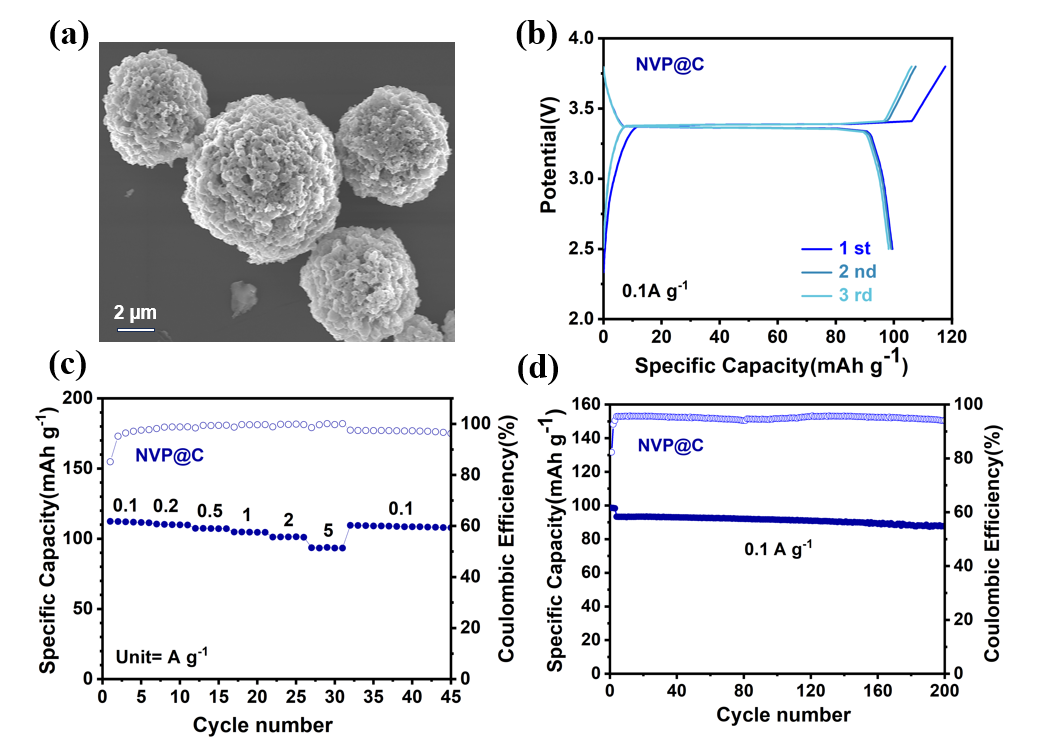


**Fig. S28 (a)** SEM images, **(b)** discharge-charge curves first three cycles, **(c)** rate capability at different current densities and **(d)** cycling performance at 0.1 A g^-1^ of the Na_3_V_2_(PO_4_)_3_@C


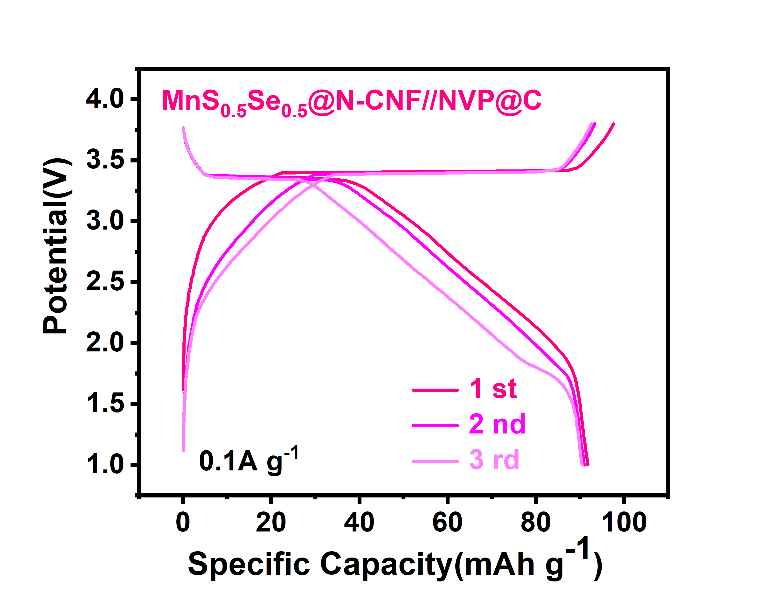


**Fig. S29** The charge/discharge curves across the ﬁrst three cycles of the full battery


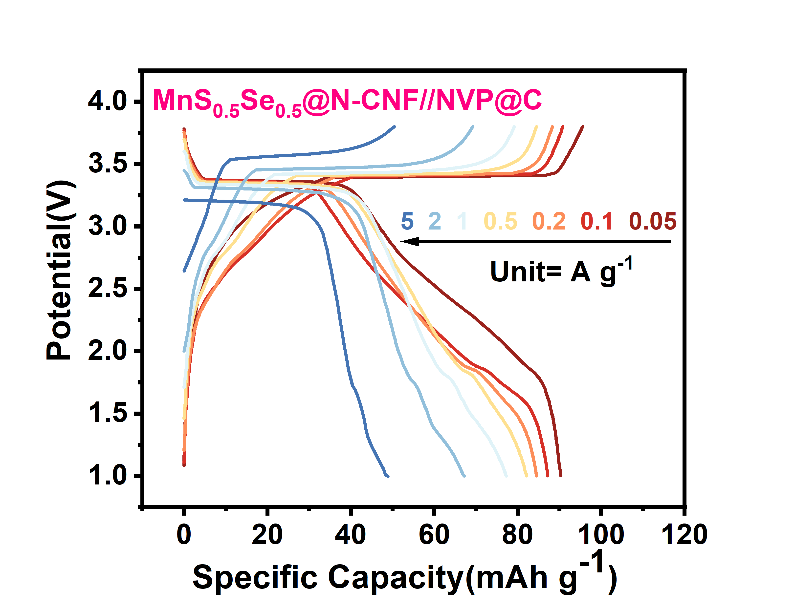


**Fig. S30** Discharging-charging profiles at different rates of the full battery

**Table S1** Comparison of the high-rate performance and long cycling stability between MnS_0.5_Se_0.5_@N-CNF and other Mn-based anode materials reported in recent literature

| Materials | Capacity/Current density | Cycle Life | Rate capability | References |
| --- | --- | --- | --- | --- |
| MnS_0.5_Se_0.5_@N-CNF | 446.8 mAh/g  (2 A/g) | 2000 cycles | 370.5 mAh/g  (10 A/g) | This  work |
| MnS@NSC | 290.0 mAh/g  (2 A/g) | 2000 cycles | 205.6 mAh/g  (10 A/g) | [S6] |
| MnS-RT | 258.6 mAh/g  (5 A/g) | 1000 cycles | 169.8 mAh/g  (10 A/g) | [S7] |
| MnS/CNTs | 225.0 mAh/g  (0.5 A/g) | 200 cycles | 170.0 mAh/g  (1 A/g) | [S8] |
| MnS/NSCTs | 411.6 mAh/g  (1 A/g) | 1400 cycles | 319.8 mAh/g  (10 A/g) | [S9] |
| MnS@N,P-LPC_2/1_ | 392.5 mAh/g  (1.6 A/g) | 1500 cycles | 392.5 mAh/g  (1.6 A/g) | [S10] |
| MnSe@C | 267.0 mAh/g  (0.5 A/g) | 1000 cycles | 209.0 mAh/g  (2 A/g) | [S11] |
| MnSe-NC | 237.0 mAh/g  (2 A/g) | 2000 cycles | 237.0 mAh/g  (2 A/g) | [S12] |
| δ-α-MnSe | 137.8 mAh/g  (1 A/g) | 1000 cycles | 81.1 mAh/g  (5 A/g) | [S13] |
| MnSe@PPyC/rGO | 346.0 mAh/g  (0.2 A/g) | 197 cycles | 294.4 mAh/g  (2 A/g) | [S14] |

**Supplementary References**

[S1] C. Lu, Z. Li, Z. Xia, H. Ci, J. Cai et al., Confining MOF-derived SnSe nanoplatelets in nitrogen-doped graphene cages via direct CVD for durable sodium ion storage. Nano Res. **12**(12), 3051-3058 (2019). <http://10.1007/s12274-019-2551-0>

[S2] X. Ren, J. Wang, D. Zhu, Q. Li, W. Tian et al., Sn-C bonding riveted SnSe nanoplates vertically grown on nitrogen-doped carbon nanobelts for high-performance sodium-ion battery anodes. Nano Energy **54**, 322-330 (2018). <http://10.1016/j.nanoen.2018.10.019>

[S3] W. Wang, P. Li, H. Zheng, Q. Liu, F. Lv et al., Ultrathin layered SnSe nanoplates for low voltage, high-rate, and long-life alkali-ion batteries. Small **13**(46), 1702228 (2017). <http://10.1002/smll.201702228>

[S4] P. Giannozzi, S. Baroni, N. Bonini, M. Calandra, R. Car et al., QUANTUM ESPRESSO: a modular and open-source software project for quantum simulations of materials. J. Phys. Condens. Matter **21**(39), 395502 (2009). <http://10.1088/0953-8984/21/39/395502>

[S5] S. Grimme, J. Antony, S. Ehrlich and H. Krieg, A consistent and accurate ab initio parametrization of density functional dispersion correction (DFT-D) for the 94 elements H-Pu. J. Chem. Phys. **132**(15), 154104 (2010). [http://10.1063/1.3382344](%20http:/10.1063/1.3382344)

[S6] S. Li, J. Chen, J. Xiong, X. Gong, J. Ciou et al., Encapsulation of MnS Nanocrystals into N, S-Co-doped Carbon as Anode Material for Full Cell Sodium-Ion Capacitors. Nano-Micro Lett. **12**(1), 34 (2020). <http://10.1007/s40820-020-0367-9>

[S7] K. Chen, G. Li, Z. Hu, Y. Wang, D. Lan et al., Construction of γ-MnS/α-MnS hetero-phase junction for high-performance sodium-ion batteries. Chem. Eng. J. **435**, 135149 (2022). [http://10.1016/j.cej.2022.135149](%20http:/10.1016/j.cej.2022.135149)

[S8] N. Zhang, X. Li, T. Hou, J. Guo, A. Fan et al., MnS hollow microspheres combined with carbon nanotubes for enhanced performance sodium-ion battery anode. Chin. Chem. Lett. **31**(5), 1221-1225 (2020). [http://10.1016/j.cclet.2019.09.050](%20http:/10.1016/j.cclet.2019.09.050)

[S9] G. Li, K. Chen, Y. Wang, Z. Wang, X. Chen et al., Cream roll-inspired advanced MnS/C composite for sodium-ion batteries: encapsulating MnS cream into hollow N,S-co-doped carbon rolls. Nanoscale **12**(15), 8493-8501 (2020). <http://10.1039/d0nr00626b>

[S10]D. Lan, Y. Zhao, Y. Liu, N. Zhu and J. Cui, Self-assembled nano-MnS@N,P dual-doped lignite based carbon as high-performance sodium-ion batteries anode. J. Energy Storage **90**, 111827 (2024). <http://10.1016/j.est.2024.111827>

[S11]J. S. Park, A. Lee, G. D. Park and Y. C. Kang, Synthesis of MnSe@C yolk‐shell nanospheres via a water vapor‐assisted strategy for use as anode in sodium‐ion batteries. Int. J. Energy Res. **46**(3), 2500-2511 (2021). <http://10.1002/er.7323>

[S12]L. Hu, L. He, X. Wang, C. Shang and G. Zhou, MnSe embedded in carbon nanofibers as advanced anode material for sodium ion batteries. Nanotechnology. **31**(33), 335402 (2020). <http://10.1088/1361-6528/ab8e78>

[S13]S. Chong, T. Li, S. Qiao, Y. C. Yang, Z. Liu et al., Boosting manganese selenide anode for superior sodium-ion storage via triggering α→β phase transition. ACS Nano **18**(4), 3801-3813 (2024). [http://10.1021/acsnano.3c12215](%20http:/10.1021/acsnano.3c12215)

[S14]N.-J. Song, Y. Wang, C. Ma, Q. Zhang, Y. Zhao et al., Cage-like MnSe@PPyC/rGO as superior dual anode materials in Li/Na-ions storage. J. Alloys Compd. **927**, 167002 (2022). [http://10.1016/j.jallcom.2022.167002](%20http:/10.1016/j.jallcom.2022.167002)
